# Macrophage NEDD4L restrains liver fibrosis by preventing scar-associated macrophage expansion via ubiquitination of phospho-SMAD3

**DOI:** 10.7150/ijbs.126649

**Published:** 2026-03-17

**Authors:** Yanghuan He, Shujun Ge, Shijia Ling, Siting Yang, Feiran Yang, Xinyi Liu, Siyue Dong, Yingfen Chen, Ziling Zhang, Yue Zhou, Seonghwan Hwang, Seung-Jin Kim, Peng Wang, Yong He, Yuanwen Chen

**Affiliations:** 1 Department of Gastroenterology, Huadong Hospital, Fudan University, Shanghai, China.; 2 School of Chinese Materia Medica, Nanjing University of Chinese Medicine, Nanjing, China.; 3 State Key Laboratory of Drug Research, Shanghai Institute of Materia Medica (SIMM), Chinese Academy of Sciences, Shanghai, China.; 4 University of Chinese Academy of Sciences, Beijing, China.; 5 Shanghai Key Laboratory of Clinical Geriatric Medicine, Shanghai Institute of Geriatrics and Gerontology, Shanghai, China.; 6 College of Pharmacy and Research Institute for Drug Development, Pusan National University, Busan, Republic of Korea.; 7 Department of Biochemistry, College of Natural Sciences, Kangwon National University, Chuncheon, Republic of Korea.; 8 Department of Hepatobiliary Medicine, Shanghai Eastern Hepatobiliary Surgery Hospital, Naval Medical University, Shanghai, China.

**Keywords:** macrophage differentiation, ubiquitination, liver fibrogenesis, p-SMAD3.

## Abstract

**Background:**

Liver fibrosis is characterized by excessive extracellular matrix deposition and hepatic stellate cell (HSC) activation, driven by chronic liver injury and inflammation. Macrophages play dual roles in fibrogenesis; the dynamic balance between pro-fibrotic and anti-fibrotic subsets is critical in determining the progression or regression of the disease. NEDD4L, an E3 ubiquitin ligase, is well-known to be involved in cell biological processes by promoting protein degradation, yet its role in macrophages and liver fibrosis remains poorly understood.

**Methods:**

Myeloid cell-specific *Nedd4l* knockout (*Nedd4l*^f/f^ Lyz-Cre+, *Nedd4l*^ΔMye^) were generated, and subjected to carbon tetrachloride (CCl_4_) and choline-deficient, L-amino acid-defined, high-fat diet (CDAHFD)-induced experimental liver fibrosis models.

**Results:**

Single-cell RNA sequencing and transcriptomic analyses revealed significant upregulation of *NEDD4L* in macrophages from human and murine fibrotic livers. Strikingly, myeloid cell-specific *Nedd4l* deficiency exacerbated liver fibrosis in both mouse models, as evidenced by increased collagen deposition and elevated expression of fibrogenic genes in *Nedd4l*^ΔMye^ mice. Notably, mice with *Nedd4l* deficient in macrophages had more pro-fibrotic scar-associated macrophage (SAM) infiltration compared with *Nedd4l*^f/f^ mice in two experimental models. More interestingly, coculture *in vitro* experiments further verified that TGF-β1-treated *Nedd4l* deficient macrophages promoted HSC activation due to greater activation of SMAD3 signaling. Mechanistically, NEDD4L directly ubiquitinated phosphorylated SMAD3 and led to its degradation, thus limiting TGF-β1/SMAD3 signaling in macrophages. Moreover, hepatic levels of NEDD4L were significantly elevated in patients with liver fibrosis, positively correlating with hepatic levels of several fibrogenic genes.

**Conclusions:**

NEDD4L serves as a critical negative regulator of liver fibrosis by restraining profibrotic SAM expansion through ubiquitination and degradation of p-SMAD3 in macrophages. These findings highlight that targeting the ubiquitin-proteasome system as a potential therapeutic strategy for the treatment of fibrotic disease.

## Introduction

Liver fibrosis, a pathological consequence of chronic liver injury caused by, e.g., viral hepatitis, alcohol abuse, or metabolic dysfunction, is characterized by excessive extracellular matrix (ECM) deposition and activation of hepatic stellate cells (HSCs) 1. Despite advances in understanding its pathogenesis, effective anti-fibrotic therapies remain limited 2, 3. Earlier studies posited that the central role of macrophages in orchestrating fibrogenesis through their dynamic polarization states—pro-inflammatory M1 macrophages driving tissue injury, while anti-inflammatory M2 macrophages promoting fibrosis. Mounting evidence demonstrate heterogeneous differentiation of hepatic macrophages in liver fibrosis, with distinct subsets exhibiting specialized functions 4, 5. Recent single-cell RNA sequencing (scRNA-seq) studies have further refined this classification, identifying novel subpopulations such as scar-associated macrophages (SAMs) and TREM2^+^ macrophages including lipid-associated macrophages (LAMs) that play decisive roles in disease progression 6, 7. However, the molecular mechanisms regulating macrophage plasticity during liver fibrogenesis are incompletely understood.

NEDD4L (neural precursor cell expressed, developmentally downregulated 4-like), a member of the NEDD4 family of E3 ubiquitin ligases, plays a vital role in regulating protein degradation and fundamental cellular processes 8. The NEDD4L protein comprises a C2 domain, three to four WW domains, and a HECT domain 9. These domains collectively mediate substrate binding and catalyze ubiquitin conjugation to target proteins, triggering their degradation via the proteasome pathway. Most identified targets of NEDD4L are membrane proteins, including ion channels and transporters 10. Recently, accumulating studies suggest that NEDD4L in hepatocytes plays an important role in the pathogenesis of metabolic dysfunction-associated steatotic liver disease (MASLD). For example, NEDD4L in hepatocytes leads to the degradation of lysosomal-associated protein transmembrane 5 (LAPTM5), which interacts with CDC42 and promotes its degradation through a lysosome-dependent manner under the stimulation of palmitic acid, finally exacerbating MASLD by activating mitogen-activated protein kinase signaling pathway 11. In addition, NEDD4L targets thioredoxin interacting protein (TXNIP) in hepatocytes, which elevates CHOP, a major regulator of ER stress-mediated apoptosis, thereby affecting the development of MASLD 12. Another interesting study found that hepatic Fas apoptotic inhibitory molecule 2 (FAIM2) is degraded by NEDD4L through the catalysis of K48-linked ubiquitination, leading to its protein downregulation. Such downregulation aggravates MASLD by promoting the autophagic degradation of CREB-regulated transcription coactivator 2 (CRTC2), a prominent regulator of lipid metabolism 13. Although these studies highlight the key role of NEDD4L in hepatocytes during MASLD progression, the functional role of NEDD4L in macrophages remains obscure.

Cytokines of the transforming growth factor-β (TGF-β) family signal through serine/threonine kinase receptors to control cellular behavior and fate 14. These signals are propagated via the transcription factors Smad2/3 downstream of TGF-β1, activin, and nodal receptors, and Smad1/5/8 downstream of bone morphogenetic protein (BMP) receptors 15. The TGF-β family of multifunctional cytokines plays a pivotal role in the pathogenesis of metabolic dysfunction-associated steatohepatitis (MASH), especially liver fibrosis development 16. More interestingly, TGF-β signaling orchestrates multiple facets of macrophage biology, including differentiation, polarization, inflammatory responses, tissue homeostasis, and tumorigenesis 17. For example, genetic disruption of TGF-β signaling compromises the development of alveolar and intestinal macrophages 18, 19. Macrophage-derived TGF-β signaling drives immunosuppression in the tumor microenvironment, facilitating immune evasion across multiple cancer types 20. Importantly, emerging evidence indicates that NEDD4L modulates the TGF-β signaling pathway by ubiquitinating key downstream substrates 21, 22. The roles of macrophage NEDD4L have been investigated in diverse pathological contexts. For instance, in viral infections, NEDD4L has been shown to promote antiviral innate immunity by catalyzing K29-linked cysteine ubiquitination of TRAF3, thereby enhancing type I interferon production 23. Conversely, in intracerebral hemorrhage (ICH)-induced brain injury, NEDD4L exacerbates neuroinflammation and brain damage by facilitating M1 macrophage polarization via the TRAF3/TBK1 signaling pathway 24. In this current study, by generating genetic knockout mouse models and ubiquinone profiling, we demonstrate that macrophage NEDD4L serves as a critical negative regulator of liver fibrosis by restraining profibrotic SAM-related macrophage expansion through ubiquitination and degradation of p-SMAD3 in macrophages. Our findings unveil novel therapeutic targets for the treatment of chronic inflammation-driven liver fibrosis by harnessing the ubiquitin-proteasome system in immune modulation.

## Materials and Methods

### Human liver samples

The paraffin-embedded human liver samples were collected from Shanghai Eastern Hepatobiliary Surgery Hospital, Naval Medical University. The study protocol received ethical approval from the hospital's Institutional Review Board (Approval No. EHBHKY2020-K-048). The baseline demographics and clinical characteristics of the patient cohorts are summarized in **Supplementary Table S1**.

### Animal models and dietary interventions

Myeloid cell-specific *Nedd4l* Knockout (*Nedd4l*^ΔMye^) mice were generated by crossing *Nedd4l* flox/flox mice with Lyz*-Cre* transgenic mice. Littermate control mice (*Nedd4l*^f/f^) were served as controls. For the carbon tetrachloride (CCl_4_)-induced liver fibrosis model, mice were intraperitoneally injected with 2 ml/kg weight of 10% CCl_4_ (MACKLIN, catalog O815211, Shanghai, China) in olive oil (MACKLIN, catalog C805325, Shanghai, China) twice per week for 4 weeks. For MASH-related liver fibrosis model, mice were fed a choline-deficient L-amino acid-defined high-fat diet (CDAHFD, A06071302; Research Diets) for 2 weeks. All animal studies were conducted under protocols approved by the Institutional Animal Care and Use Committee (IACUC) of the Shanghai Institute of Materia Medica, Chinese Academy of Sciences and the Institutional Animal Care and Use Committee of Fudan University (IACUC-2023-012). Mice were housed under a 12-hours light/dark cycle with *ad libitum* access to food and water.

### Differentiation of primary bone marrow-derived macrophages (BMDMs)

Irrigate the bone marrow cavity of the mouse femur and tibia with sterile PBS using a syringe, then grind the tissue and filter it through a 70-μm cell strainer. Next, the samples were centrifuged at 1600 rpm, 4 °C, for 5 minutes to precipitate the bone marrow cells (BM). The red blood cells were lysed using red blood cell lysis buffer, resuspended cells in PBS, and filtered through a 70-μm cell strainer. BM cells were seeded into 24-well cell culture plates and added conditioned medium containing 20 ng/ml MCSF (1640 medium, 10% FBS + 1% PS), and cultured in a 37 °C, 5% CO₂ incubator. On day 3, add the same fresh conditioned medium and continue culturing. After day 7, the cells were washed with PBS and used for subsequent experiments.

### Plasmid construction

The pCDH-CMV-HIS-UB-MCS-3Flag-EF1-CopGFP-T2A-Puro plasmid was generated by inserting the UB into the XbaI/AsisI sites of the multicoloning site (MCS) of the pCDH vector backbone. The plasmid was commercially synthesized and subcloned by YiXueSheng Biosciences Inc (Shanghai, China). The final construct was validated through double restriction enzyme digestion analysis and full-length sequencing using primers specific to the CMV promoter.

The pcDNA3.1-SMAD3-Myc plasmid was generated by inserting the SMAD3 into the BamHI/EcoRI and pcDNA3.1sites of the multicoloning site (MCS) of the pCDH vector backbone. The plasmid was commercially synthesized and subcloned by YiXueSheng Biosciences Inc (Shanghai, China). The final construct was validated through double restriction enzyme digestion analysis and full-length sequencing using primers specific to the CMV promoter and the bGh terminator.

The pcDNA3.1-Nedd4l-Flag plasmid was generated by inserting the NEDD4L into the BamHI/EcoRI and pcDNA3.1sites of the multicoloning site (MCS) of the pCDH vector backbone. The plasmid was commercially synthesized and subcloned by WeiZhenSheng Biosciences Inc (Shanghai, China). The final construct was validated through double restriction enzyme digestion analysis and full-length sequencing using primers specific to the CMV promoter and the bGh terminator.

### *In vitro* co-culture assays

BMDMs were differentiated as described above and then stimulated with or without 10 ng/mL TGF-β1 for 24 hours. The culture supernatant was then removed, and the cells were washed and incubated in serum-free DMEM for another 12 hours to generate conditioned medium (CM). The CM was then collected, centrifuged to remove cellular debris, and mixed at a 1:1 ratio with fresh RPMI-1640 medium. This mixture was applied to pre-plated, unactivated LX-2 hepatic stellate cells (HSCs). Co-cultures were maintained for various durations, as indicated, after which cells and/or supernatants were harvested for subsequent analysis.

### Biochemical assays

Serum alanine aminotransferase (ALT) and aspartate aminotransferase (AST) levels were determined using ALT Detection Kit (Nanjing Jiancheng Bioengeering Institute, China, catalog C009-3-1) and AST Detection Kit (Nanjing Jiancheng Bioengeering Institute, China, catalog C010-3-1) according to the manufacturer's instruction.

### Immunohistochemistry and Sirius red staining

For Sirius red staining, the paraffin-embedded sections were dewaxed in xylene and stained with PICRO-RED STAINING SOLUTION (Phygene, catalog 20230310, Fuzhou, China) for 1 hour. For immunohistochemistry, sections were subjected to antigen retrieval with citrate buffer (Invitrogen, catalog 005000, CA, USA). After incubating at 3% H_2_O_2_, sections were blocked with 3% normal goat serum buffer (NGS). Then, the sections were incubated with primary antibodies overnight at 4 °C and incubated with secondary antibodies (Cell Signaling Technology, Danvers, MA, catalog 8814S or 8125S) at room temperature for 1 hour. ImmPACT DAB Substrate Kit (Vector Laboratories, catalog ZK1018, CA, USA) or ImmPACT Red Substrate Kit (Vector Laboratories, catalog ZJ1205, CA, USA) were used to visualize the staining. Primary antibodies used were listed below: F4/80 (Cell Signaling Technology catalog 70076S, Danvers, MA, USA), Myeloperoxidase (Biocare medical, catalog APR023AA, CA, USA), CD3 (Cell Signaling Technology catalog 78588S, Danvers, MA, USA), α-SMA (Cell Signaling Technology, catalog 19245S, Danvers, MA, USA), Col1α1 (Cell Signaling Technology, catalog 72026S, CST, Danvers, MA), NEDD4L (Cell Signaling Technology, catalog 4013, Danvers, MA, USA), p-Smad3 (ABclonal Technology, catalog AP1263, Wuhan, China), FABP5 (ABclonal Technology, catalog A27255, Wuhan, China), CD9 (ABclonal Technology, catalog A19027, Wuhan, China), CD163 (ABclonal Technology, catalog A8383, Wuhan, China), CD206 (ABclonal Technology, catalog A21014, Wuhan, China), TLR1 (ABclonal Technology, catalog A0997, Wuhan, China), Trem2 (Abcam, catalog AB305103 Cambridge, UK). Images were obtained using the Olympus BX43 microscope.

### Immunofluorescence

Fresh liver tissues were frozen and embedded for sectioning. After overnight fixation with 4% PFA at 4 °C, 3% NGS was used to block for one hour at room temperature. Next, primary antibodies were incubated overnight at 4°C, and secondary antibodies (Alexa Fluor 488 or 555 goat anti-rabbit IgG [H+L], Cell Signaling Technology, catalog 4412S or 4413S; Alexa Fluor 488 or 555 goat anti-mouse IgG [H+L], Cell Signaling Technology, catalog 4408S or 4409S, Danvers, MA, USA) were incubated for 1 hour at room temperature. TrueVIEW Autofluorescence Quenching Kit (Vector Laboratories, catalog ZK0818, CA, USA) was used to eliminate non-specific fluorescence. Nuclear staining was performed using 4', 6'-diamino-2-phenylindole (DAPI) (Beyotime, catalog P0131, Shanghai, China). Images were captured with Olympus APEXVIEW APX100 microscope. The following primary antibodies were used: NEDD4L (Cell Signaling Technology, catalog 4013, Danvers, MA, USA), IBA1 (catalog No. MABN92, Merck KGaA, Darmstadt, Germany), CD206 (ABclonal Technology, catalog A21014, Wuhan, China), FABP5 (ABclonal Technology, catalog A27255, Wuhan, China).

### Total RNA isolation and reverse transcription quantitative PCR (RT-qPCR)

Total RNA was extracted using RNA isolater Total RNA Extraction Reagent (Vazyme, catalog 7E0131K3, Nanjing, China), and single-stranded cDNA was synthesized using the High-Capacity cDNA Reverse Transcription Kit (Thermo Fisher Scientific, catalog 2816898, Waltham, MA). Gene expression was determined by qPCR using ChamQ Universal SYBR qPCR Master Mix (Vazyme, catalog 7E751K3, Nanjing, China) and a QuantStudio 5 Instrument (Thermo Fisher Scientific, Waltham, MA). The levels of the rRNA 18s were used as an internal control. Each test was performed in triple replication and the 2^-∆∆Ct^ method was used to calculate the expression of mRNA. The primers used for RT-qPCR are listed in **Supplementary Table 2**.

### Co-immunoprecipitation (Co-IP) assay

Co-immunoprecipitation assays were performed to investigate the interaction between [Protein A] and [Protein B]. Briefly, HEK293T cells transfected with relevant plasmids were lysed in IP lysis buffer (20 mM Tris-HCl, pH 7.5, 150 mM NaCl, 1% NP-40, 10% glycerol) supplemented with a protease inhibitor cocktail (Thermo Fisher Scientific, catalog 88804, Waltham, MA, USA) on ice for 5 minutes. The lysates were centrifuged for 10 minutes at 4 °C to remove cellular debris. The supernatant was then incubated with 2 μg of Mouse anti DDDDK-Tag (ABclonal Technology, catalog AE005, Wuhan, China) or Myc-Tag Rabbit mAb (ABclonal Technology, catalog AE070, Wuhan, China) overnight at 4 °C with gentle rotation. Subsequently, 25 μL of Protein A/G Magnetic Beads (Thermo Fisher Scientific, catalog 88804, Waltham, MA, USA) were added and incubated for an additional 1 hour at 4 °C. The beads were then washed five times with ice-cold lysis buffer. Finally, the bound proteins were eluted by boiling in 2× Laemmli SDS sample buffer at 95 °C for 10 minutes. The collected protein samples were used for subsequent experiments.

### Western blotting

Proteins were extracted from liver tissues or cells using RIPA Buffer (Thermo Fisher Scientific, catalog YH374135, Waltham, MA, USA) containing Halt Protease and Phosphatase Inhibitors (Thermo Fisher Scientific, catalog 78447, Waltham, MA, USA). The samples were loaded into polyacrylamide gels (Absin, catalog 220A019, Shanghai, China) and then transferred onto nitrocellulose membranes (Merck, catalog 0000208128, Darmstadt, Germany). The nitrocellulose membranes were blocked with 1% BSA and incubated with antibodies overnight at 4 °C. After incubation with anti-rabbit or anti-mouse IgG HRP-linked second antibody (Cell Signaling Technology, catalog 7074S or 7076S, Danvers, MA, USA), protein bands were visualized with SuperSignal Maximum Sensitivity Substrate (Thermo Fisher Scientific, catalog WG328673, Waltham, MA, USA). The following antibodies were used: Col1α1 (Abcam, catalog AB260043, Cambridge, UK,), α-SMA (Thermo Fisher Scientific, catalog 14-9760-82, Waltham, MA, USA), NEDD4L (Cell Signaling Technology, catalog 4013, Danvers, MA, USA), p38 (Cell Signaling Technology, catalog 8690S, Danvers, MA, USA), p-P38 (Cell Signaling Technology, catalog 4511, Danvers, MA, USA), ERK (Cell Signaling Technology, catalog 4695S, Danvers, MA, USA), p-ERK (Cell Signaling Technology, catalog 4370S, Danvers, MA, USA), p-Smad2/3 (Cell Signaling Technology, catalog 8828S, Danvers, MA, USA), Smad2/3 (Cell Signaling Technology, catalog 8685S, Danvers, MA, USA), p-Smad3 (ABclonal Technology, catalog AP1263, Wuhan, China), HRP-conjugated Mouse anti-Rabbit IgG Light Chain (ABclonal Technology, catalog AS061, Wuhan, China), Mouse anti DDDDK-Tag (ABclonal Technology, catalog AE005, Wuhan, China), HA-Tag Rabbit mAb (ABclonal Technology, catalog AE105, Wuhan, China), Myc-Tag Rabbit mAb (ABclonal Technology, catalog AE070, Wuhan, China).

### Statistical analysis

Data are expressed as the mean ± SEM and were analyzed using GraphPad Prism software (version 9.4.1; GraphPad Software). To compare values obtained from two groups, the Student *t* test was performed; values from multiple groups were compared using one-way ANOVA. *p<*0.05 was considered significant.

## Result

### Macrophage NEDD4L is elevated during liver fibrosis development

**Figure 1:**
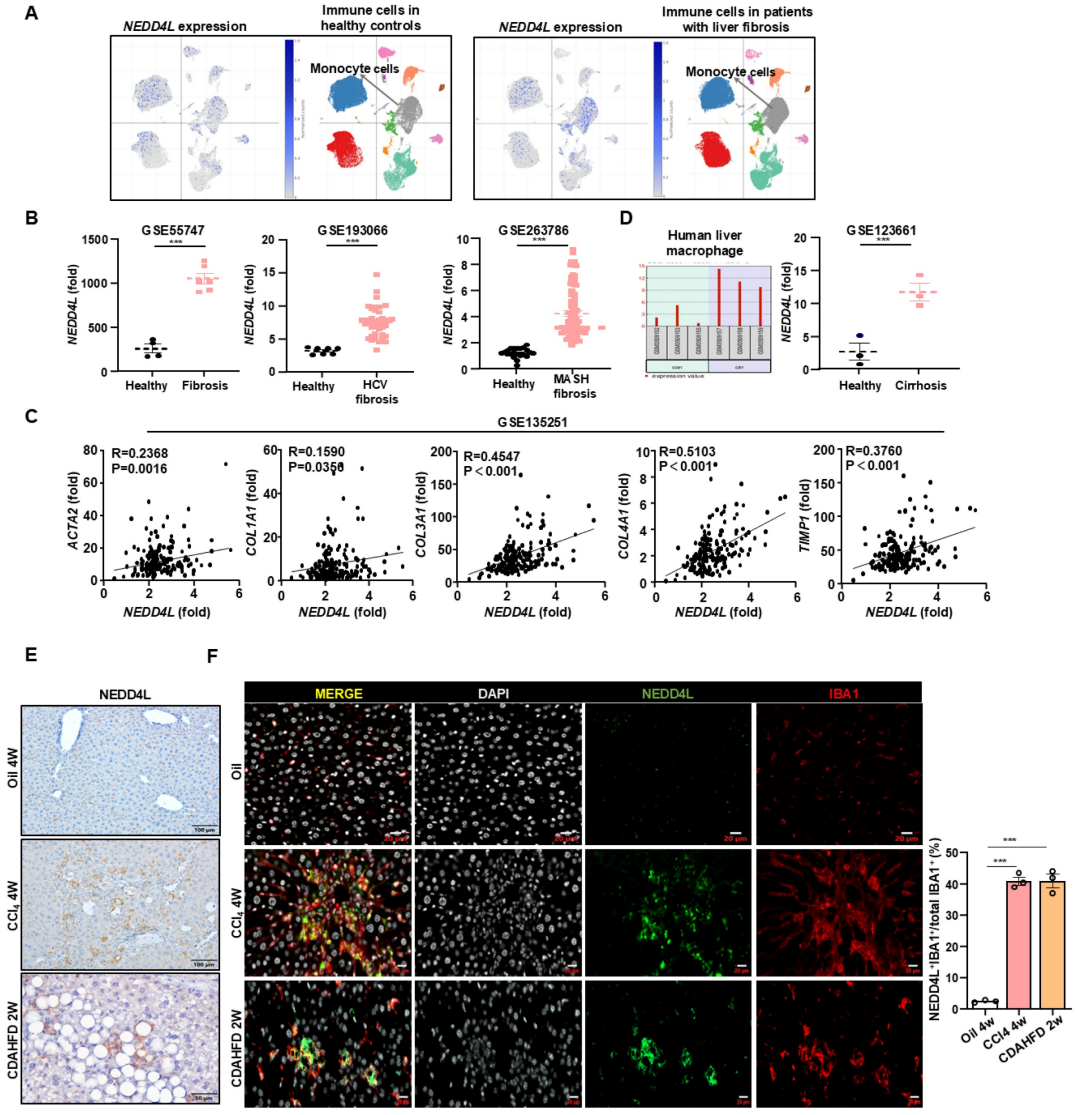
** Macrophage NEDD4L is upregulated in human and murine liver fibrosis. (A)** scRNA-seq analysis showing *NEDD4L* expression in immune cells from healthy and fibrotic human livers. **(B)** Cross-dataset validation of NEDD4L upregulation in HCV- and MASLD-induced fibrosis (GSE55747, GSE193066, and GSE263786). **(C)** The correlations between hepatic *NEDD4L* levels and liver fibrogenic genes in patients with liver fibrosis were analyzed from the GSE135251 dataset. **(D)** *NEDD4L* expression in isolated hepatic macrophages from cirrhosis patients (GSE123661). **(E)** Representative images of NEDD4L staining (Scale bar: 200 μm) in the livers of control and liver fibrosis mouse models are shown. **(F)** Representative immunofluorescence staining of IBA1 (red), NEDD4L (green), nuclei (white) (Scale bar: 20 μm) in the livers of CCl_4_ and CDAHFD-induced liver fibrosis mice are shown. The percentage of positive cells was quantified. Values represent mean ± SEM. ***p<0.001.

To understand the role of NEDD4L in liver fibrosis, we first analyzed public single-cell RNA sequencing (scRNA-seq) database (https://singlecell.broadinstitute.org). Initial scRNA-seq analysis of healthy human livers revealed only minimal Nedd4l expression across immune cell populations. In contrast, analysis of samples from patients with liver fibrosis showed a marked induction of NEDD4L. Such upregulation was observed in several immune populations, and was most pronounced within monocyte-derived macrophages (Fig. 1A, Supporting Fig. S1A). This striking, cell-type-associated induction indicates a disease-specific transcriptional reprogramming, likely reflecting the functional adaptation of this myeloid subset during liver fibrogenesis. To assess the generality of this response, we further performed a cross-database analysis of transcriptomic datasets from fibrotic livers of diverse etiologies. Consistent upregulation of *NEDD4L* was observed in HCV-induced fibrosis (GSE193066) and metabolic dysfunction-associated steatotic liver disease (MASLD)-driven fibrosis (GSE263786) compared to healthy controls (Fig. 1B). In the MASLD fibrosis dataset (GSE135251), *NEDD4L* expression showed a positive correlation with several core pro-fibrotic genes including *ACTA2*, *COL1A1*, *COL3A1*, *COL4A1*, and *TIMP1*, suggesting a potential relationship between NEDD4L and liver fibrogenesis (Fig. 1C). Moreover, analysis of isolated hepatic macrophages from cirrhotic patients confirmed elevated *NEDD4L* expression at the cellular levels, reinforcing its link to advanced disease (Fig. 1D). This induction was conserved across species, as elevated NEDD4L was also observed in murine models of carbon tetrachloride (CCl_4_)-induced and choline-deficient, L-amino acid-defined high-fat diet (CDAHFD)-induced liver fibrosis (Fig. 1E). In fibrotic livers induced by both CCl_4_ and CDAHFD, immunofluorescence co-staining revealed a specific population of IBA1⁺ macrophages within the fibrotic lesions. Critically, NEDD4L expression was predominantly co-localized with these cells, which collectively established the myeloid cell-specific upregulation of NEDD4L during liver fibrosis (Fig. 1F). Taken together, these multi-level analyses—spanning single-cell RNA sequencing, cross-dataset validation, molecular correlation, and protein-level visualization—consistently demonstrate that NEDD4L expression is significantly upregulated in hepatic macrophages in both human and experimental mouse liver fibrosis, highlighting its potential role as a key regulator in the fibrotic microenvironment.

### Myeloid cell-specific knockout of *Nedd4l* exacerbates CCl_4_-induced liver fibrosis

**Figure 2:**
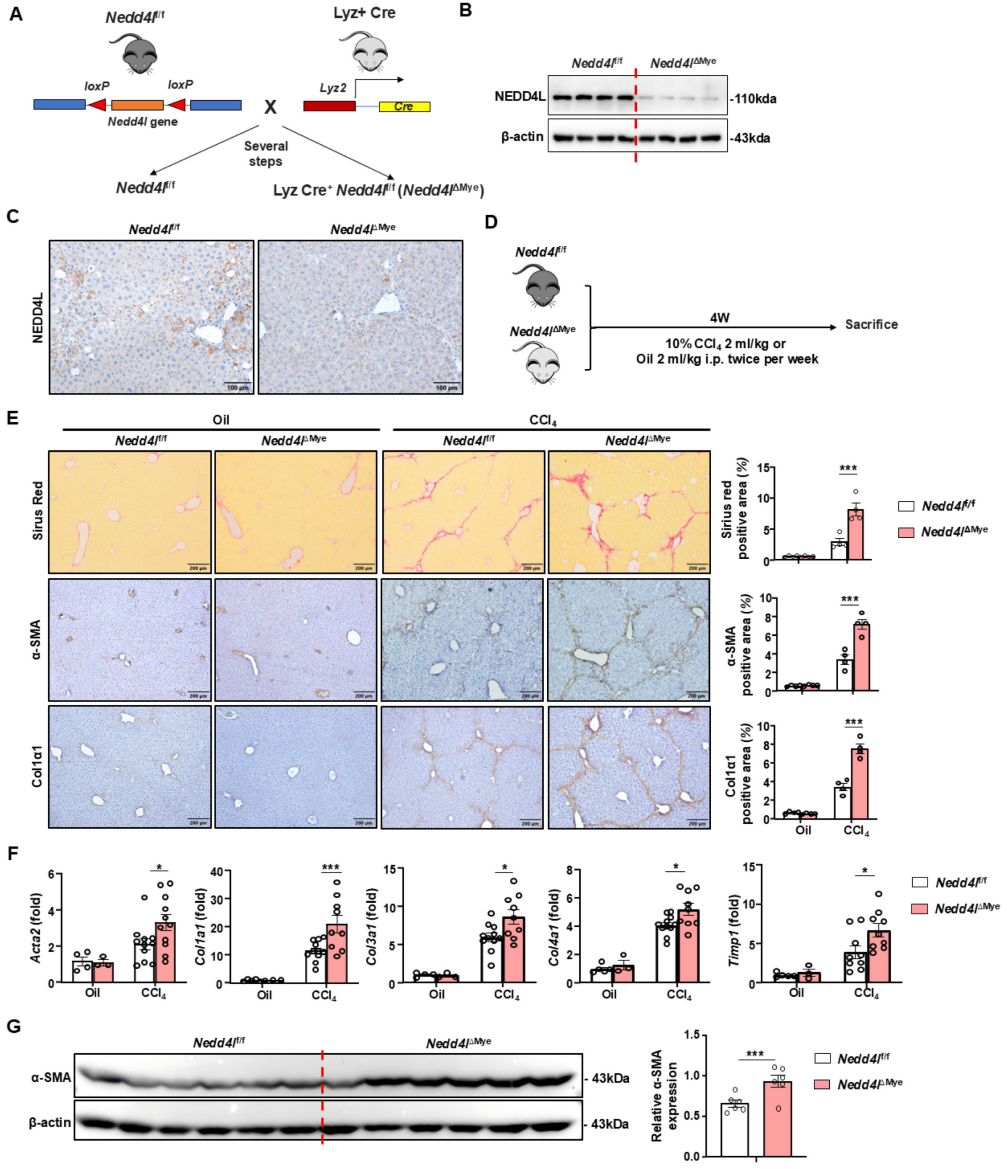
** Myeloid cell-specific knockout of *Nedd4l* exacerbates CCl_4_-induced liver fibrosis. (A)** Strategy for generating myeloid cell-specific *Nedd4l* knockout mice.** (B-C)** Validation of *Nedd4l* knockout efficiency by western blot in BMDMs and by IHC staining in liver sections. **(D)** Eight-week-old male *Nedd4l*^ΔMye^ mice and their *Nedd4l*^f/f^ littermate controls were subjected to CCl_4_ or olive oil injection for 4 weeks. **(E)** Representative images of Sirius red staining (Scale bar: 200 μm), α-SMA staining (Scale bar: 200 μm) and Col1α1 staining (Scale bar: 200 μm) from *Nedd4l*^f/f^ and *Nedd4l*^ΔMye^ mice are shown. The percentage of positive area was quantified. **(F)** The hepatic expression of *Acta2*, *Col1a1*, *Col3a1*, *Col4a1* and *Timp1* were analyzed by RT-qPCR in liver tissues from *Nedd4l*^f/f^ and *Nedd4l*^ΔMye^ mice. **(G)** Protein levels of α-SMA in liver tissues were assessed by Western blotting. β-actin was used as a loading control. Values represent mean ± SEM. *p<0.05, ***p<0.001.

To clarify the role of NEDD4L in liver fibrosis, we constructed myeloid cell-specific *Nedd4l* knockout mice (*Nedd4l*^ΔMye^) (Fig. 2A). The knockout efficiency was validated using Western blot by isolating bone marrow-derived macrophages (Fig. 2B) and immunofluorescence (Fig. 2C, Supporting Fig. S1B), confirming significantly reduced NEDD4L expression in macrophages compared to littermate controls. To induce liver fibrosis, both *Nedd4l*^ΔMye^ and their *Nedd4l*^fl/fl^ counterparts were subjected to a 4-week regimen of intraperitoneal injections of CCl_4_ or olive oil (vehicle control) (Fig. 2D). Myeloid cell-specific *Nedd4l* deficiency exacerbated liver fibrosis as demonstrated that *Nedd4l*^ΔMye^ mice had greater collagen deposition and elevated α-smooth muscle actin (α-SMA) (Fig. 2E). While no significant fibrotic changes or intergroup differences were observed in the vehicle-treated mice, CCl_4_-treated *Nedd4l*^ΔMye^ mice displayed substantially expanded fibrotic areas. Consistent with these morphological findings, RT-qPCR analysis further demonstrated that hepatic expression of several key profibrogenic genes including *Acta2, Col1a1, Col3a1, Col4a1*, and *Timp1,* was significantly elevated in *Nedd4l*^f/f^ mice compared to vehicle controls (Fig. 2F). Subsequent western blot validation confirmed a robust elevation in α-SMA protein expression in CCl_4_-treated *Nedd4l*^ΔMye^ mice compared to that in *Nedd4l*^f/f^ counterparts (Fig. 2G). Collectively, these data indicate that myeloid cell-specific deletion of *Nedd4l* aggravates CCl_4_-induced experimental mouse liver fibrosis.

### Myeloid cell-specific knockout of *Nedd4l* worsens CDAHFD-induced liver fibrosis

**Figure 3:**
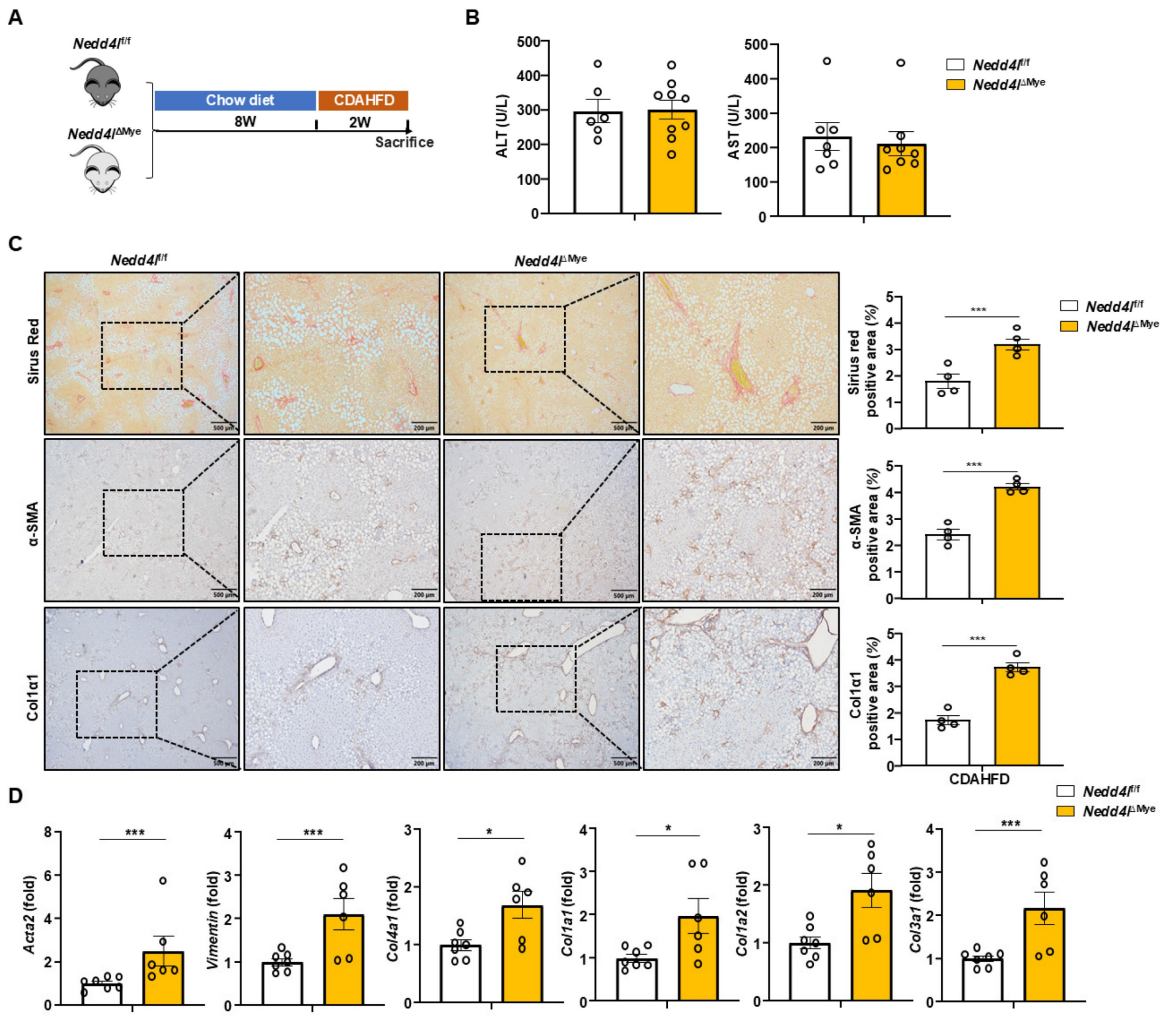
***Nedd4l* deficiency in macrophages aggravates CDAHFD-induced liver fibrosis. (A)** Eight-week-old male *Nedd4l*^ΔMye^ mice and their littermate *Nedd4l*^fl/f^ mice were fed CDAHFD for 2 weeks to establish the experimental MASH-related liver fibrosis model. **(B)** Serum ALT and AST levels were measured.** (C)** Representative images of Sirius red staining (Scale bar: 200 μm), α-SMA staining (Scale bar: 200 μm) and Col1α1 staining (Scale bar: 200 μm) from *Nedd4l*^f/f^ and *Nedd4l*^ΔMye^ mice are shown. The percentage of positive area was quantified. **(D)** The hepatic expression of *Acta2*, *Vimentin, Col4a1*, *Col1a1*, *Col1a2* and *Col3a1* were analyzed by RT-qPCR in liver tissues from *Nedd4l*^f/f^ and *Nedd4l*^ΔMye^ mice. Values represent mean ± SEM. *p<0.05, ***p<0.001.

To define the role of macrophage NEDD4L in nutrition-induced hepatic fibrosis, we employed a well-established dietary model using a CDAHFD-induced liver fibrosis model, which recapitulates key aspects of metabolic dysfunction-associated steatohepatitis (MASH) and progressive fibrogenesis 25 (Fig. 3A). Following two weeks of CDAHFD feeding, serum ALT and AST levels were comparable between *Nedd4l*^ΔMye^ mice and their *Nedd4l*^f/f^ littermate controls (Fig. 3B), suggesting comparable initial liver damage under this short-term nutritional challenge. Interestingly, *Nedd4l*^ΔMye^ mice exhibited substantially expanded fibrotic areas along with significantly intensified deposition of α-SMA⁺ and Col1α1⁺ cells (Fig. 3C). RT-qPCR profiling demonstrated remarkable upregulation of a panel of fibrogenic genes in the livers of *Nedd4l*^ΔMye^ mice including *Acta2, Col1a1, Col3a1, Col4a1,* and* Vim* (Fig. 3D), indicating myeloid cell-specific Nedd4l deficiency also exacerbated CDAHFD-induced liver fibrosis. The accelerated fibrosis in *Nedd4l*^ΔMye^ mice after only two weeks of CDAHFD feeding suggested a critical role for macrophage NEDD4L in restraining the early fibrogenic response. To validate this function in a distinct, rapid model of monocyte-driven fibrosis, we employed bile duct ligation (BDL) model (Supporting Fig. S2A). Immunofluorescence analysis confirmed NEDD4L expression in liver macrophages following BDL (Supporting Fig. S2B). Consistent with our previous findings, *Nedd4l*^ΔMye^ mice developed greater liver fibrosis after BDL compared to *Nedd4l*^f/f^ littermates as evidenced by increased collagen deposition (Supporting Fig. S2C), and by elevated mRNA expression of several key fibrotic markers (e.g., *Col1a1, Acta2*) as well as pro-fibrogenic genes in macrophages (e.g., *Fabp5, Gpnmb*) (Supporting Fig. S2D), underscoring the generalizable role of NEDD4L in suppressing the fibrogenic activity of macrophages. The consistent exacerbation across both nutritional and toxic injury paradigms underscores a central protective role for macrophage NEDD4L in restraining liver fibrogenesis, independent of the original insult, and highlights its potential as a cross-disease modulator of liver fibrosis.

### Myeloid cell-specific knockout of *Nedd4l* promotes scar-associated macrophage (SAM) differentiation in liver fibrosis

Extensive evidence indicate that macrophages contribute to liver fibrosis through diverse mechanisms. They are highly plastic and can adopt various activation states, broadly categorized into two major phenotypes: classically activated (M1) pro-inflammatory macrophages and alternatively activated (M2) macrophages, which are often associated with tissue repair and pro-fibrotic responses 26. To understand whether immune cell infiltration was involved in exacerbated liver fibrosis in *Nedd4l*^ΔMye^ mice, we performed immunohistochemical staining of F4/80, MPO and CD3. Unexpectedly no significant differences in the number of F4/80⁺ macrophages, MPO⁺ neutrophils, and CD3⁺ T cells were observed between *Nedd4l*^ΔMye^ and *Nedd4l*^f/f^ mice (Supporting Fig. S3A). RT-qPCR corroborated these findings: although CCl_4_ challenge significantly upregulated several key inflammatory genes including *Il1b, Il6, Tnfa, Mmp1, Cxcl1,* and* Ccl2* in *Nedd4l*^f/f^ mice, hepatic expression of these genes did not differ significantly between *Nedd4l*^ΔMye^ and *Nedd4l*^f/f^ groups (Supporting Fig. S3B). Serum ALT and AST levels were similarly elevated following CCl₄ administration, without significant differences between* Nedd4l*^ΔMye^ and *Nedd4l*^f/f^ mice, and baseline levels of serum ALT and AST were also comparable between untreated groups, indicating that myeloid-specific deletion of *Nedd4l* does not cause spontaneous hepatocellular injury (Supporting Fig. S3C).This pattern was consistently observed in the CDAHFD nutritional model as well, with no intergroup differences in F4/80⁺ or MPO⁺ immune cell densities (Supporting Fig. S4A) or in the expression of inflammatory genes such as *Il1b, Tnfa*, and *Ccl2* (Supporting Fig. S4B). These findings clearly demonstrated that myeloid cell-specific deletion of *Nedd4l* exacerbates liver fibrosis possibly through inflammation-independent mechanisms.

**Figure 4:**
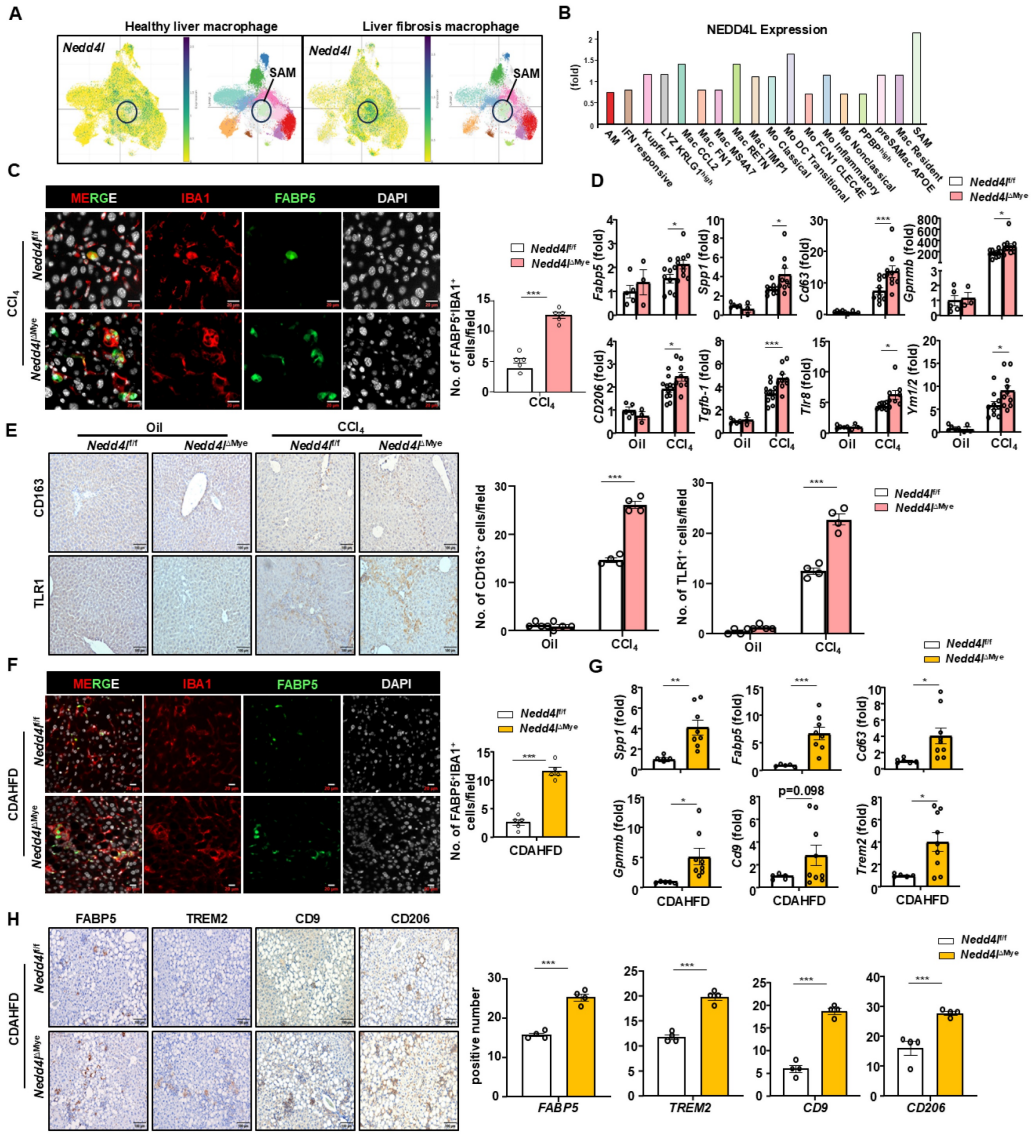
** Myeloid cell-specific knockout of *Nedd4l* promotes scar-associated macrophage (SAM) differentiation during liver fibrosis. (A)** Analysis of *Nedd4l* expression in liver macrophages from patients with liver fibrosis by analyzing single-cell sequencing database (Liver Cell Atlas: https://singlecell.broadinstitute.org). **(B)** Analysis of *Nedd4l* expression in SAMs from patients with liver fibrosis by analyzing liver single-cell sequencing database (Liver Cell Atlas: https://singlecell.broadinstitute.org). **(C)** Representative immunofluorescence images of IBA1 (red), FABP5 (green), and DAPI (white) (Scale bar: 10 μm) in the livers of CCl_4_-treated *Nedd4l*^f/f^ and* Nedd4l*^ΔMye^ mice. The number of positive cells was quantified. **(D)** The hepatic expression of SAM-related profibrotic genes were analyzed by RT-qPCR in liver tissues from oil or CCl_4_-treated* Nedd4l*^f/f^ and *Nedd4l*^ΔMye^ mice.** (E)** Representative images of CD163 staining (Scale bar: 100 μm) and TLR1 staining (Scale bar: 200 μm) from CCl_4_-treated* Nedd4l*^f/f^ and *Nedd4l*^ΔMye^ mice are shown. The number of positive cells was quantified. **(F)** Representative immunofluorescence images of IBA1 (red), FABP5 (green), and DAPI (white) (Scale bar: 10 μm) in the livers of CDAHFD-fed *Nedd4l*^f/f^ and* Nedd4l*^ΔMye^ mice are shown. The number of positive cells was quantified. **(G)** The hepatic expression of SAM-related profibrotic genes were analyzed by RT-qPCR in liver tissues from CDAHFD-fed* Nedd4l*^f/f^ and *Nedd4l*^ΔMye^ mice.** (H)** Representative images of FABP5 staining (Scale bar: 100 μm), TREM2 staining (Scale bar: 100 μm), CD9 staining (Scale bar: 100 μm), and CD206 staining (Scale bar: 100 μm) from CDAHFD-fed* Nedd4l*^f/f^ and *Nedd4l*^ΔMye^ mice are shown. The number of positive cells was quantified. Values represent mean ± SEM. *p < 0.05, **p < 0.01, ***p < 0.001.

Given the absence of a discernible inflammatory effect, we turned our attention to another macrophage subset critically involved in liver fibrogenesis: pro-fibrotic Trem2^+^FABP5^+^SPP1^+^CD9^+^ scar-associated macrophages (SAMs), which have been implicated in hepatic injury repair and activation of hepatic stellate cells through production of TGF-β1—a process that, when dysregulated, directly exacerbates fibrosis^6^, ^27^. Single-cell transcriptomic analysis of human fibrotic livers revealed that SAMs exhibited markedly elevated *Nedd4l* expression compared to negligible levels in healthy controls (https://singlecell.broadinstitute.org/single_cell) (Fig. 4A-B). Furthermore, these fibrotic SAMs concurrently highly expressed pro-fibrotic mediators including *CCL22*, VEGFA, and *TLR1* (Supporting Fig. S5A). More importantly, immunofluorescence staining and quantitative cell counting revealed a significant increase in FABP5⁺, a specific marker for SAMs, in *Nedd4l*^ΔMye^ livers compared to controls (Fig. 4C). Consistent with this finding, co-staining for IBA1⁺CD206⁺ macrophages similarly revealed a significant expansion in this population (Supporting Fig. S5B), suggesting an overall increase in the SAM-profibrogenic macrophage compartment in *Nedd4l*^ΔMye^ mice. RT-qPCR analysis further confirmed upregulation of SAM signature genes including *Spp1, Cd63, Gpnmb,* and* Fabp5* as well as pro-fibrotic mediators including *Mrc1, Chil3 (Ym1), Tlr8, and Tgfb1* in CCl_4_-treated *Nedd4l*^ΔMye^ mice (Fig. 4D). In addition, the expression of pro-fibrotic macrophage markers (TLR1 and CD206) was greater in *Nedd4l*^ΔMye^ mice than *Nedd4l*^f/f^ controls after CCl_4_ injection (Fig. 4E). These results suggested that *Nedd4l* deficiency in macrophages promotes SAM differentiation, thereby accelerating toxin-induced liver fibrosis progression.

Consistent with the findings from the CCl_4_-induced liver fibrosis model, immunofluorescence staining of murine fibrotic livers also showed elevated IBA1⁺FABP5⁺ macrophages in *Nedd4l*^ΔMye^ mice compared to *Nedd4l*^f/f^ controls after CDAHFD feeding (Fig. 4F). Subsequent RT-qPCR analysis corroborated these results, revealing pronounced upregulation of SAM-associated transcripts including *Spp1, Cd63, Trem2, Gpnmb*, and Fabp5, in *Nedd4l*^ΔMye^ livers with *Cd9* showing a consistent upward trend (Fig. 4G). Immunohistochemical analysis of liver sections from mice fed CDAHFD for two weeks showed significantly elevated expression of SAM markers (TREM2, CD9, FABP5) and pro-fibrotic macrophage marker CD206 in *Nedd4l*^ΔMye^ mice compared to *Nedd4l*^fl/fl^ controls (Fig. 4H). Notably, the pro-fibrotic polarization of *Nedd4l*-deficient macrophages was not attributable to altered cell survival or a generalized hyper-inflammatory state, as their viability and classic inflammatory response to lipopolysaccharide (LPS) remained comparable to controls (Supporting Fig. S6A). Taken together, these data from both toxin-induced and nutrition-based fibrotic models demonstrate that myeloid cell-specific knockout of *Nedd4l* potentiates SAM differentiation and pro-fibrotic activation independently of classical inflammatory pathways.

### NEDD4L limits pro-fibrotic macrophage phenotype via regulation of SMAD3 phosphorylation

**Figure 5:**
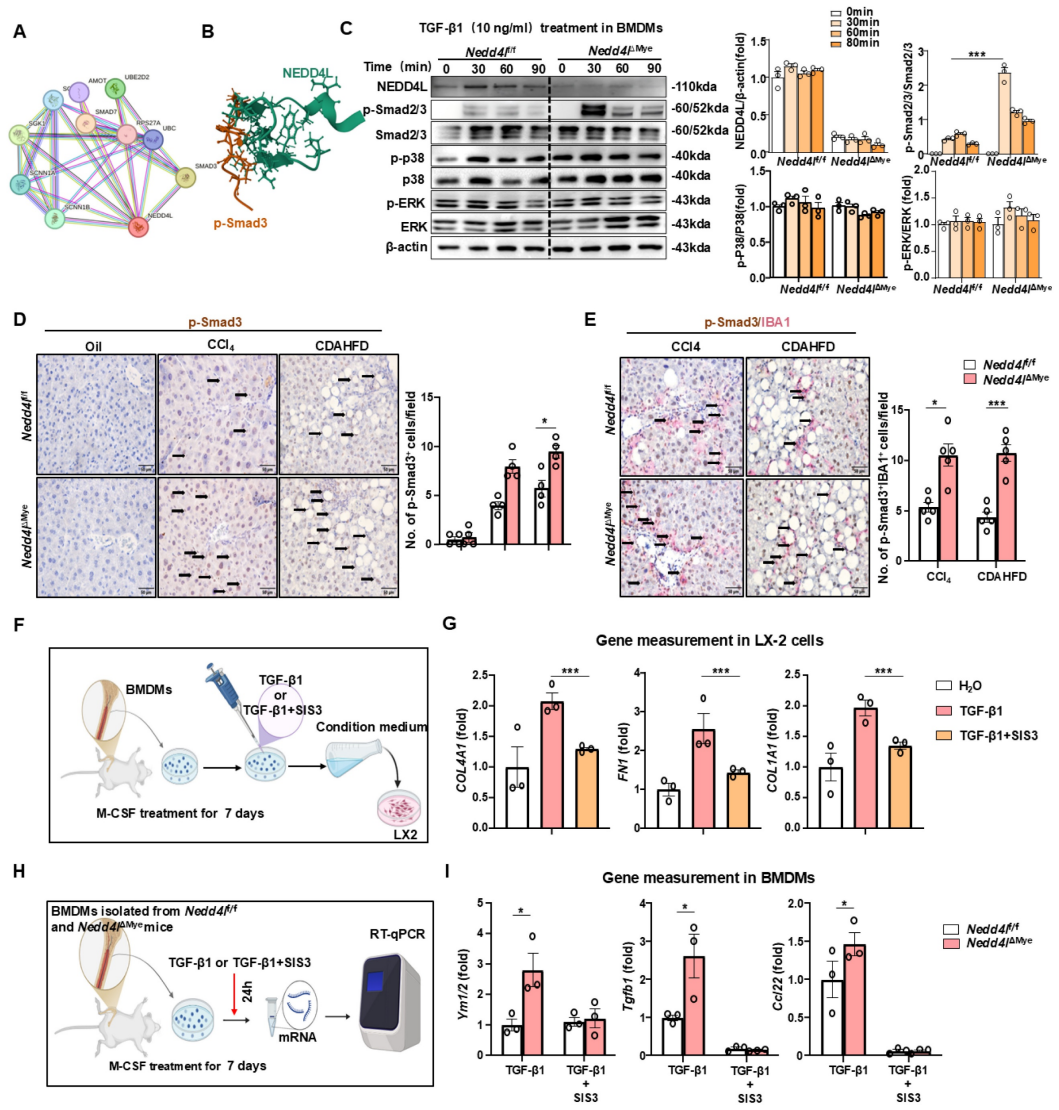
** NEDD4L limits pro-fibrotic macrophage polarization via inhibiting SMAD3 phosphorylation. (A)** Protein interaction network analysis (https://cn.string-db.org). **(B)** Structural prediction of the interaction between NEDD4L and phosphorylated SMAD3 protein (https://www.rcsb.org/).** (C)** BMDMs isolated from *Nedd4l*^f/f^ and *Nedd4l*^ΔMye^ mice were stimulated with M-CSF (20 ng/ml) for 7 days, and then stimulated with 10 ng/ml TGF-β1. Western blot analysis of TGF-β1 signal pathway. β-actin was used as a loading control. **(D)** Representative images of p-Smad3 staining (Scale bar: 50 μm) from oil-treated, CCl_4_- treated and CDAHFD-fed *Nedd4l*^f/f^ and *Nedd4l*^ΔMye^ mice are shown. The number of positive cells was quantified. **(E)** Representative images of p-Smad3 and IBA1 (Scale bar: 50 μm) double staining of liver tissue sections from CCl_4_-treatedand CDAHFD-fed *Nedd4l*^f/f^ and *Nedd4l*^ΔMye^ mice are shown. The number of positive cells was quantified. **(F)** Schematic diagram of experimental design. BMDMs isolated from C57BL/6J mice were differentiated with M-CSF (20 ng/ml, 7 days) prior to a 24-hour stimulation with TGF-β1 (10 ng/ml), with or without SIS3 (5 μM). Conditional BMDM medium were co-cultured with LX-2 cells. **(G)** The expression of fibrogenic genes were analyzed by RT-qPCR in LX-2 cells after culture with conditional medium from BMDMs. **(H)** Schematic diagram of experimental design. BMDMs isolated from *Nedd4l*^f/f^ and *Nedd4l*^ΔMye^ mice were differentiated with M-CSF (20 ng/ml, 7 days) prior to a 24-hour stimulation with TGF-β1 (10 ng/ml), with or without SIS3 (5 μM). **(I)** The expression of pro-fibrotic genes was analyzed by RT-qPCR in BMDMs after culture with different conditional medium. Values represent mean ± SEM. *p< 0.05, ***p < 0.001.

To elucidate the mechanisms underlying post-TGF-β1 responses in macrophages, we first performed protein-protein interaction analysis (https://cn.string-db.org), which identified NEDD4L as a key regulator of SMAD3, suggesting a potential role in modulating TGF-β1 signaling activity (Fig. 5A). As an E3 ubiquitin ligase, NEDD4L primarily exerts its biological functions by regulating target proteins through ubiquitination. Computational structural analysis of ubiquitin ligase-substrate interactions predicted that NEDD4L directly bound phosphorylated SMAD3 (https://www.rcsb.org) (Fig. 5B). Subsequent molecular profiling demonstrated that myeloid cell-specific Nedd4l knockout significantly potentiates SMAD2/3 activation as evidenced that TGF-β1 stimulation resulted in markedly enhanced phosphorylation of SMAD2/3 (p-SMAD2/3) in macrophages from *Nedd4l*^ΔMye^ mice compared to that from *Nedd4l*^f/f^ controls, while total SMAD2/3 protein levels remained unchanged (Fig. 5C). Importantly, this effect was specific to the canonical TGF-β pathway, as non-canonical pathways including ERK and p38 MAPK signaling were unaltered (Fig. 5C). Importantly, this enhanced TGF-β1 responsiveness in *Nedd4l*-deficient macrophages was specific, as their viability and classical inflammatory response to LPS treatment were unaltered (Supporting Fig. S6B).

Next, immunohistochemistry of liver sections revealed a substantially more prominent infiltration of p-SMAD3⁺ immune cells in fibrotic livers than that in control tissues (Fig. 5D). To this end, immunohistochemical double staining was performed. A notably expanded population of IBA1⁺/p-SMAD3⁺ macrophages was readily detectable in *Nedd4l*^ΔMye^ fibrotic livers compared to *Nedd4l*^f/f^ controls (Fig. 5E), these observed increases were confirmed by quantitative analysis, providing histological evidence that *Nedd4l* deficiency in macrophages enhances SMAD3 activation specifically in macrophages under fibrotic conditions.

To investigate the contribution of macrophage p-SMAD3 to HSC activation, we collected conditioned medium from the pharmacological SMAD3 inhibitor SIS3-treated macrophages (5mM refreshed every 12h for 48h) primary bone marrow-derived macrophages (BMDMs) (Fig. 5F). This conditioned medium significantly attenuated the activation of LX-2 cells as indicated by reduced expression of *TGFB1*, *FN1* and *COL4A1* (Fig. 5G). Pharmacological inhibition of p-SMAD3 with SIS3 obviously diminished the pro-fibrotic gene signature (*Ym1/2, Tgfb1, Ccl22*) in BMDMs from *Nedd4l*^ΔMye^ mice, thereby functionally establishing the dependence of this enhanced fibrogenic phenotype on p-SMAD3 signaling (Fig. 5H-I). These comprehensive data suggest that *Nedd4l* deficiency in macrophages drives pro-fibrotic polarization primarily through enhanced p-SMAD3-dependent signaling mechanisms, which in turn promotes HSC activation and contributes to fibrosis pathogenesis.

**Figure 6:**
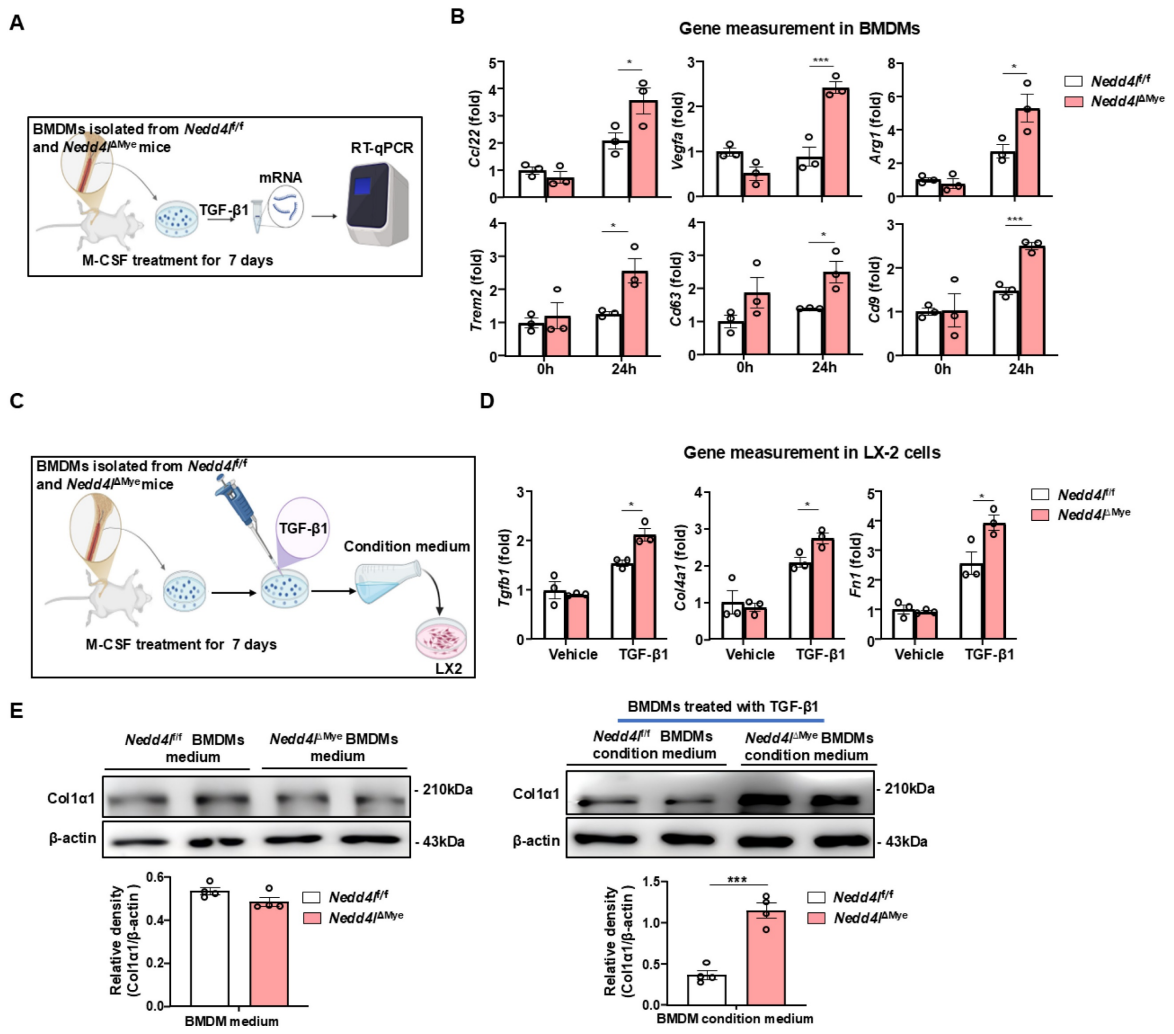
** Myeloid cell-specific knockout of *Nedd4l* enhances TGF-β1-induced pro-fibrotic macrophage polarization *in vitro*. (A)** Schematic diagram of experimental design. BMDMs isolated from *Nedd4l*^f/f^ and *Nedd4l*^ΔMye^ mice were stimulated with M-CSF (20 ng/ml) 7 days, and then stimulated with 10 ng/ml TGF-β1 for 24 hours. **(B)** The expression of pro-fibrotic genes was analyzed by RT-qPCR in BMDMs. **(C)** Schematic diagram of experimental design. BMDMs isolated from *Nedd4l*^f/f^ and *Nedd4l*^ΔMye^ mice were stimulated with M-CSF (20 ng/ml) 7days, and then stimulated with 10 ng/ml TGF-β1 for 24 hours. Conditional BMDM medium were co-cultured with LX-2 cells. **(D)** The expression of fibrogenic genes was analyzed by RT-qPCR in LX-2 cells treated with conditional medium from *Nedd4l*^f/f^ and *Nedd4l*^ΔMye^ macrophages.** (E)** Protein levels of Col1α1 in LX-2 cells treated with conditional medium from *Nedd4l*^f/f^ and *Nedd4l*^ΔMye^ macrophages were assessed by Western blotting. β-actin was used as a loading control. Values represent mean ± SEM. *p< 0.05, ***p< 0.001.

Given that TGF-β1 participates in pro-fibrotic macrophage differentiation 28, we sought to further elucidate the mechanistic impact of macrophage *Nedd4l* deficiency on this process through a comprehensive *in vitro* approach. Primary BMDMs were differentiated and employed to model physiological conditions *in vitro*. BMDMs were differentiated with M-CSF for 7 days to attain a mature macrophage phenotype, followed by TGF-β1 stimulation for 24 hours (Fig. 6A). RT-qPCR analysis demonstrated that under basal conditions, the expression of SAM macrophage-related genes (*Trem2, Cd9,* and *Cd63*) and pro-fibrogenic genes (*Arg1, Ccl2,* and *Vegfa*) did not differ significantly between unstimulated BMDMs from *Nedd4l*^f/f^ and BMDMs from* Nedd4l*^ΔMye^. However, upon TGF-β1 stimulation for 24 hours, BMDMs from *Nedd4l*^ΔMye^ mice exhibited a markedly greater amplitude of induction in the expression of these genes compared to that from *Nedd4l*^fl/fl^ controls (Fig. 6B). These results indicate that *Nedd4l* deficiency in macrophages predisposes macrophages toward a pro-fibrogenic SAM phenotype.

To determine whether the secretory phenotype of *Nedd4l*-deficient macrophages functionally activated HSCs, we established a simplified co-culture system. Unactivated LX-2 human HSCs were treated with conditioned medium collected from BMDMs, with or without exogenous TGF-β1 supplementation (Fig. 6C). LX-2 cells exposed to conditioned medium from *Nedd4l*^ΔMye^ BMDMs in the presence of TGF-β1 exhibited significantly higher expression of several key fibrogenic genes, including *TGFB1*, *COL4A1*, *and FN1* (Fig. 6D). Western blot analysis further confirmed elevated protein levels of Col1α1 in LX-2 cells incubated with *Nedd4l*^ΔMye^ macrophage-conditioned medium (Fig. 6E), providing the compelling evidence that TGF-β1-treated *Nedd4l* deficient macrophages are more prone to promote HSC activation compared with TGF-β1-treated control macrophages. Collectively, these *in vitro* data suggest that *Nedd4l* deficiency in macrophages not only intrinsically enhances their pro-fibrotic transcriptional response to TGF-β1 but also augments their capacity to activate HSCs via paracrine mechanisms. To further validate these findings and address potential species differences, we performed the key co-culture experiments using the murine HSC cell line JS-1 cells. Conditioned medium from *Nedd4l*^ΔMye^ BMDMs similarly enhanced the expression of fibrogenic genes (*Acta2, Col1a1*) in JS-1 cells, corroborating our results in a species-matched system (Supporting Fig. S7).

### NEDD4L mediates ubiquitination of phosphorylated SMAD3, leading to its degradation

**Figure 7:**
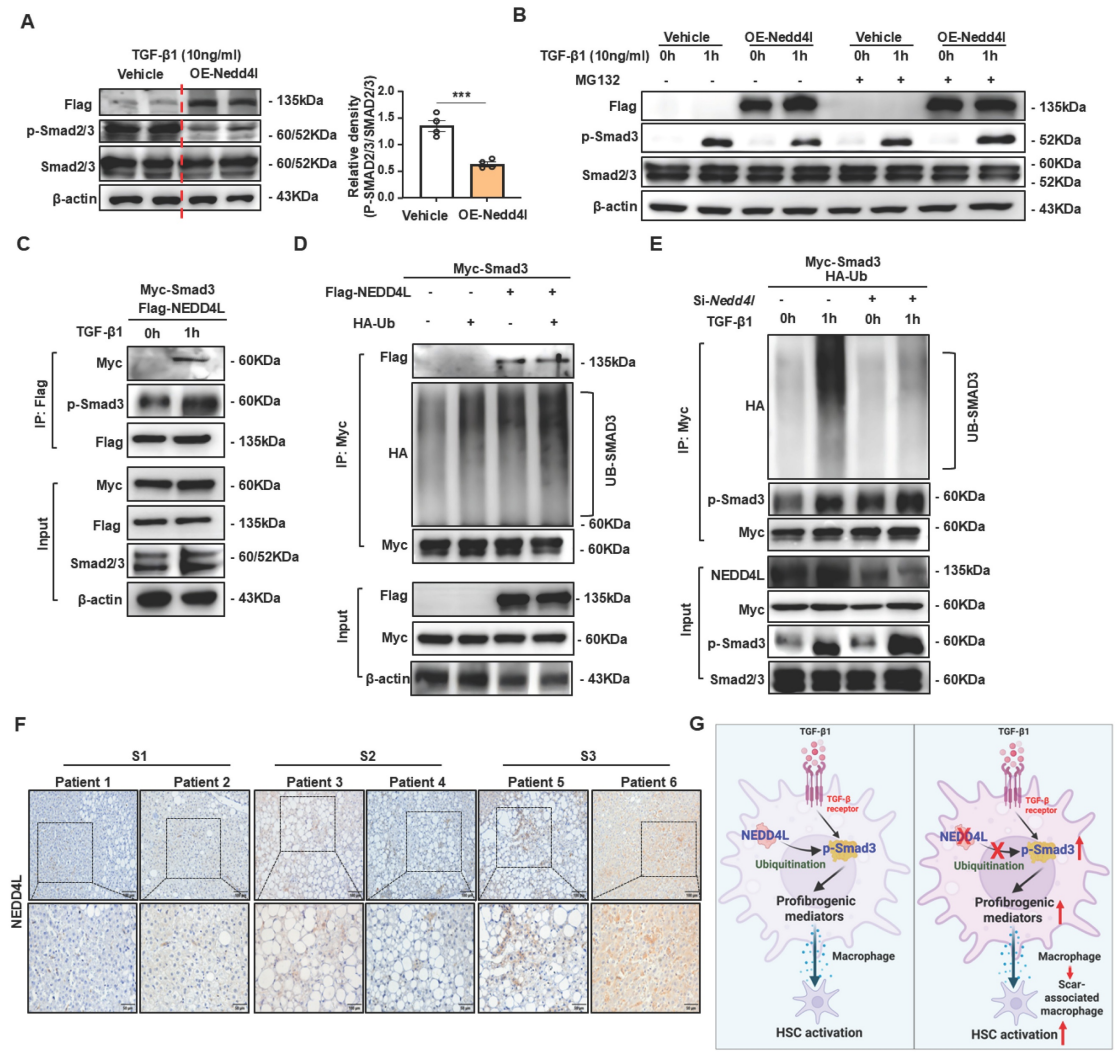
** NEDD4L promotes ubiquitination of phosphorylated SMAD3, leading to its degradation. (A)** HEK293T cells were transfected with NEDD4L overexpression or control plasmid for 24 hours prior to TGF-β1 stimulation. Protein levels were assessed by Western blotting.** (B)** HEK293T cells were transfected with NEDD4L or control plasmid for 24 hours prior to a 1-hour TGF-β1 stimulation with or without MG132. Protein levels were assessed by Western blotting.** (C)** Co-immunoprecipitation of Myc-Smad3 with Flag-NEDD4L from the transfected HEK293T cell lysates using an anti-Flag antibody, followed by western blot analyses. **(D)** HEK293T cells were co-transfected with HA-tagged ubiquitin, Flag-Nedd4L, and Myc-Smad3. Myc immunoprecipitates from whole-cell lysates were analyzed by immunoblotting with the indicated antibodies. **(E)** HEK293T cells were transfected with NEDD4L or control siRNA for 24 hours prior to HA-ubiquitin plasmid transfection. After another 24 hours, the cells were stimulated with TGF-β1 for 1 hour. Smad2/3 immunoprecipitates and whole-cell lysates were then analyzed by immunoblotting. **(F)** Liver tissues of patients with different stage of liver fibrosis were collected. Representative images of NEDD4L staining (Scale bar: 200 μm and 50 μm) are shown.** (G)** Schematic model of NEDD4L-p-SMAD3 axis in macrophages during liver fibrosis. TGF-β1 signaling initiates pro-fibrotic response in macrophages by inducing p-SMAD3-mediated SAM differentiation. Concurrently, NEDD4L elevation in macrophages forms a negative feedback circuit to limit liver fibrosis by promoting p-SMAD3 degradation. Values represent mean ± SEM. ***p<0.001.

To investigate whether NEDD4L directly ubiquitinates phosphorylated SMAD2/3, we conducted a series of well-controlled experiments in HEK293T cell lines. First, we transfected HEK293T cells with a NEDD4L-Flag overexpression plasmid, stimulated them with TGF-β1 for 1 hour, and analyzed the cell lysates by Western blot. As shown in Fig. 7A, NEDD4L overexpression significantly reduced the protein levels of phosphorylated SMAD2/3 (p-SMAD2/3) without altering total SMAD2/3 expression, suggesting a specific regulatory effect on the activated form of these transcription factors. To determine whether this reduction was mediated through ubiquitination-dependent proteasomal degradation, we performed TGF-β1 stimulation in the presence of MG132, a potent proteasome inhibitor. MG132 treatment effectively rescued the decrease in phosphorylated SMAD2/3 levels induced by NEDD4L overexpression (Fig. 7B), supporting that NEDD4L promotes proteasomal degradation of p-SMAD2/3.

We next examined whether NEDD4L directly interacted with p-SMAD3. HEK293T cells were co-transfected with SMAD3-Myc and NEDD4L-Flag plasmids, stimulated with TGF-β1, and then subjected to co-immunoprecipitation (co-IP) using Flag antibodies. As shown in Fig. 7C, p-SMAD3-Myc was specifically co-precipitated with NEDD4L-Flag only upon TGF-β1 stimulation. Critically, p-SMAD2/3 was detected in these immunoprecipitates, confirming that NEDD4L binds directly to p-SMAD3 in a TGFβ-dependent manner.

To further evaluate whether this interaction leads to ubiquitination of p-SMAD3, we co-transfected HEK293T cells with Ub-HA and SMAD3-Myc, with or without NEDD4L-Flag. Following TGF-β1 stimulation and immunoprecipitation of SMAD3-Myc, we observed significantly enhanced ubiquitination of p-SMAD3 in the presence of NEDD4L (Fig. 7D). Conversely, when endogenous NEDD4L was knocked down using shRNA targeting NEDD4L (shNEDD4L), TGF-β1-induced ubiquitination of p-SMAD3 was markedly attenuated (Fig. 7E), providing additional genetic evidence for the essential role of NEDD4L in this process. Taken together, these results demonstrate that NEDD4L directly binds to phosphorylated SMAD3 upon TGFβ stimulation and promotes its ubiquitination and subsequent proteasomal degradation. This mechanism maintains tight control over the duration and intensity of TGFβ signaling.

To investigate the clinical correlation and significance of NEDD4L, we assembled a cohort of human liver tissues from patients with fibrosis of multiple etiologies and across a spectrum of disease severity. Immunohistochemistry staining of NEDD4L showed that NEDD4L protein expression was elevated in human severe fibrotic liver tissues compared with mild fibrotic tissues (Fig. 7F).

## Discussion

NEDD4L, a member of the NEDD4 family of E3 ubiquitin ligases, is known to regulate protein degradation and affect various cellular processes, including TGF-β signaling 22. However, its cell type-specific functions, particularly in macrophages during the development of liver fibrosis, remain poorly understood. In this study, we demonstrate that myeloid cell-specific deletion of *Nedd4l* exacerbates liver fibrosis by promoting SAM-related macrophage differentiation via enhancing TGF-β/SMAD3 signaling in macrophages. These findings uncover a critical role for macrophage NEDD4L in restraining liver fibrogenesis and highlight its potential as a therapeutic target for the treatment of liver fibrosis (Fig. 7G).

Although NEDD4L has been implicated in various pathological contexts, including viral infection 23, brain injury 24, organ fibrosis 29, 30, and cancers 31, 32, its function in liver fibrosis—particularly within macrophages—has not been previously elucidated. A key mechanistic insight from our study is the identification of p-SMAD3 as a direct substrate of NEDD4L in macrophages. Previous reports indicated that NEDD4L can ubiquitinate SMAD2/3 and TGF-β receptors 33, but its role in modulating SMAD3 stability in macrophages was unknown. We showed that TGF-β1 stimulation promoted the physical interaction between NEDD4L and p-SMAD3, leading to its ubiquitination and degradation. Crucially, NEDD4L specifically targets activated, not resting-state, Smad2/3 for degradation. This exquisite selectivity requires a two-step verification: first, TGF-β1-induced activation, and second, CDK8/9-mediated phosphorylation of a specific pT-PY motif that is recognized by the WW2 domain of NEDD4L. This specific mechanism ensures precise termination of TGF-β signaling without disturbing the basal cellular pool of Smad2/3 22. In the absence of NEDD4L, p-SMAD3 accumulates and drives sustained expression of pro-fibrotic genes, thereby accelerating fibrosis. This places NEDD4L as a central negative regulator of TGF-β signaling in macrophages, operating at the level of downstream SMAD activation. Notably, the profibrotic effect of myeloid NEDD4L deficiency was not primarily mediated by amplifying classic inflammation, as evidenced by unaltered or even reduced expression of cytokines including *Il6* and *Tnfa* in fibrotic *Nedd4l*^ΔMye^ livers. This indicates that NEDD4L specifically restrains a fibrogenesis-prone macrophages, which can be dissociated from the canonical pro-inflammatory response. Although NEDD4L has been reported to ubiquitinate SMAD2 in certain contexts 34, our results also revealed that *Nedd4l* deficiency attenuates the ubiquitin-mediated degradation of p-SMAD2, leading to its accumulation in our liver fibrosis models (data not shown). However, as previous studies suggest that SMAD2 has a minimal impact on macrophage differentiation 35. The potential non-canonical roles of SMAD2 in macrophages present an intriguing subject for future research. Our data indicate that its predominant fibro-suppressive function in hepatic macrophages is mediated through SMAD3. While both SMAD2 and SMAD3 are key effectors of TGF-β1 signaling, they often play non-redundant roles, with SMAD3 being particularly critical in driving pro-fibrotic gene expression. It is worth noting that the basal turnover of p-SMAD3 is primarily mediated by the ubiquitin-proteasome system, with several E3 ligases—including SMURF1/2 33, 34—implicated in this process. The classical negative feedback regulator SMAD7 36, which is induced by TGF-β signaling, is known to recruit SMURF E3 ligases to target both TGF-β receptors and activated SMAD2/3 complexes for degradation. In contrast, our data suggest that NEDD4L exhibits more selective activity towards p-SMAD3 in macrophages, potentially operating independently of SMAD7-mediated recruitment. This specificity is therapeutically crucial, as global inhibition of SMAD degradation through central regulators like SMAD7 may cause widespread unintended consequences, whereas targeting the more discrete NEDD4L-pSMAD3 interaction offers a unique opportunity for cell-type and substrate-selective intervention in fibrotic diseases.

A critical question arising from our findings is how the accumulation of p-SMAD3, resulting from *Nedd4l* deletion in macrophages, precisely orchestrates the phenotypic transition of macrophages towards a pro-fibrotic SAM state. We hypothesized that sustained SMAD3 activation initiates a transcriptional reprogramming that extends beyond classical TGF-β1 targets. Specifically, the prolonged nuclear retention of p-SMAD3 may directly bind to and activate promoters of key pro-fibrotic mediators, creating an autocrine loop that perpetuates the activated state of SAMs. Furthermore, p-SMAD3 likely collaborates with other transcription factors and epigenetic modifiers to establish a stable pro-fibrotic gene expression signature. Supporting this notion, the anti-fibrotic drug pirfenidone (PFD) has been shown to ameliorate fibrosis in radiation-induced lung injury (RILI) primarily by suppressing p-Smad3, which subsequently attenuates M2 macrophage infiltration. This inhibition subsequently attenuates M2 macrophage infiltration and disrupts the downstream activation of the TGF-β1/Smad3 pathway 37. Through these mechanisms, SAMs are known to actively promote hepatic fibrogenesis by sustaining TGF-β secretion, producing pro-fibrotic mediators such as PDGF and CTGF, directly activating hepatic stellate cells, and inhibiting ECM degradation—collectively driving the progression of liver fibrosis 38, 39. The exact repertoire of genes directly regulated by the NEDD4L-p-SMAD3 axis in SAMs and its crosstalk with other signaling pathways represents a vital area for future investigation, as it will provide deeper insights into the molecular drivers of macrophage plasticity in fibrosis.

Another significant finding is the role of NEDD4L in restraining SAM differentiation. SAMs have recently emerged as key contributors to fibrosis progression 39. Our data show that *Nedd4l* deficiency in macrophages drives SAM expansion and enhances their pro-fibrotic output, suggesting that NEDD4L acts as an intrinsic brake on macrophage phenotypic switching toward a profibrotic state. From a clinical perspective, targeting NEDD4L may offer a novel therapeutic strategy for liver fibrosis. Unlike broad TGF-β inhibitors 40, which often cause systemic side effects such as immune suppression or cardiovascular complications, enhancing NEDD4L activity could provide a more specific means of dampening fibrogenic signaling within the liver microenvironment. Mechanistically, the role of NEDD4L may extend beyond SMAD3 regulation. As an E3 ubiquitin ligase, NEDD4L likely has multiple substrates in macrophages that contribute to its anti-fibrotic function. For example, NEDD4L is known to regulate membrane receptors and transporters that could influence macrophage migration, cytokine secretion, or cross-talk with other hepatic cells 33. Additionally, the WW domains of NEDD4L mediate interactions with proline-rich motifs present in many signaling proteins 8, suggesting possible involvement in other pathways such as Wnt/β-catenin 41 or Notch 42, which are also implicated in fibrogenesis. The specific upstream regulators of NEDD4L in fibrotic macrophages—such as cytokines, metabolic signals, or mechanical stress—also warrant further investigation, as they may reveal additional targets for intervention.

In conclusion, our study uncovers macrophage NEDD4L as a crucial regulator of liver fibrosis through ubiquitin-mediated degradation of p-SMAD3 and suppression of SAM differentiation. These findings not only advance our understanding of the ubiquitin-proteasome system in immune-mediated fibrogenesis but also identify NEDD4L as a potential target for anti-fibrotic therapy.

## Supplementary Material

Supplementary figures and tables.

## Abbreviations

Acta2: Actin Alpha 2, Smooth Muscle
ALT: Alanine Aminotransferase
ARG1/Arg1: Arginase 1
AST: Aspartate Aminotransferase
BMDM: Bone Marrow-Derived Macrophage
CCl_4_: Carbon Tetrachloride
CCL2: C-C Motif Chemokine Ligand 2
CCL22: C-C Motif Chemokine Ligand 22
CDAHFD: Choline-deficient, L-amino acid-defined, High-fat Diet
Col1α1: Collagen Type I Alpha 1 Chain
Col3α1: Collagen Type III Alpha 1 Chain
Col4α1: Collagen Type IV Alpha 1 Chain
Co-IP: Co-Immunoprecipitation
CXCL1: C-X-C Motif Chemokine Ligand 1
DAPI: 4′,6-Diamidino-2-Phenylindole
ECM: Extracellular Matrix
ERK: Extracellular Signal-Regulated Kinase
FABP5/Fabp5: Fatty Acid Binding Protein 5
GPNMB: Glycoprotein Nmb
HECT: Homologous to the E6-AP Carboxyl Terminus
HSC: Hepatic Stellate Cell
IBA1: Ionized Calcium Binding Adaptor Molecule 1
IHC: Immunohistochemistry
IL1β: Interleukin 1 Beta
IL-6: Interleukin 6
M-CSF: Macrophage Colony-Stimulating Factor
MAPK: Mitogen-Activated Protein Kinase
MASH: Metabolic dysfunction-associated steatohepatitis
MASLD: Metabolic dysfunction-associated steatotic liver disease
MPO: Myeloperoxidase
MRC1: Mannose Receptor C-Type 1 (CD206)
NEDD4L: Neural precursor cell expressed, developmentally down-regulated 4-like
p-ERK: Phosphorylated ERK
p-SMAD2/3: Phosphorylated SMAD2/3
p-p38: Phosphorylated p38 MAPK
PBS: Phosphate Buffered Saline
RT-qPCR: Quantitative Reverse Transcription Polymerase Chain Reaction
SAM: Scar-Associated Macrophage
scRNA-seq: Single-Cell RNA Sequencing
shRNA: Short Hairpin RNA
siRNA: Small Interfering RNA
SPP1/Spp1: Secreted Phosphoprotein 1 (Osteopontin)
TGF-β1: Transforming Growth Factor Beta 1
TIMP1: Tissue Inhibitor of Metalloproteinases 1
TLR1/8: Toll-Like Receptor 1/8
TNFα: Tumor Necrosis Factor Alpha
TREM2: Triggering Receptor Expressed on Myeloid cells 2
VEGFA: Vascular Endothelial Growth Factor A
VIM: Vimentin
