# Supplementary material for: Macrophage NEDD4L restrains liver fibrosis by preventing scar-associated macrophage expansion via ubiquitination of phospho-SMAD3
Source: Int J Biol Sci. 2026 Mar 17;22(7):3304–21. doi: 10.7150/ijbs.126649 (PMC13085682; doi:10.7150/ijbs.126649)
Supplement: Supplementary file 1 — Supplementary figures and tables. [file ijbsv22p3304s1.pdf]

Supporting Figure S1

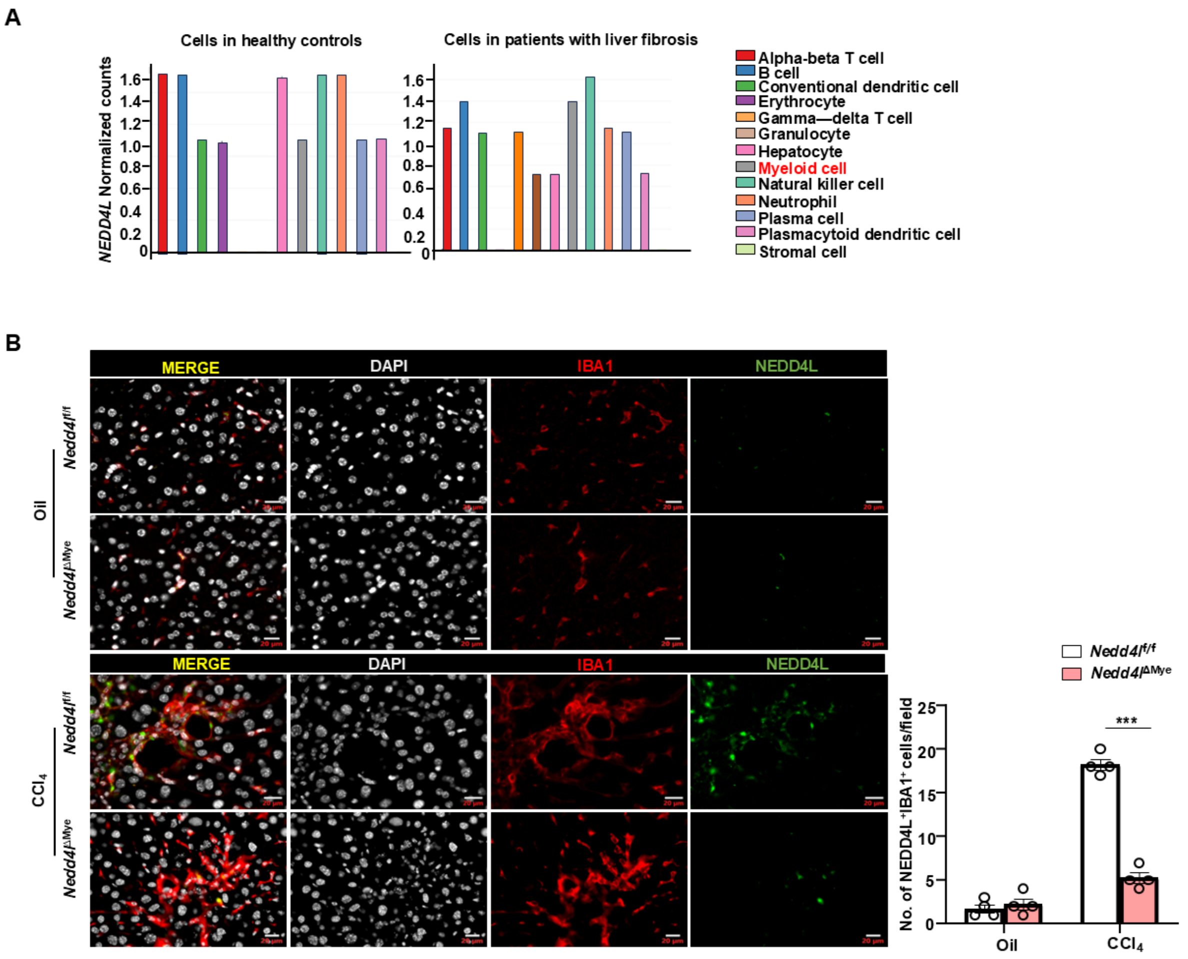

Supporting Figure S1. NEDD4L is mainly expressed in monocyte-derived macrophages. (A) Analysis of *Nedd4l* expression in Liver cells from healthy and patients with liver fibrosis by analyzing liver single-cell sequencing database (Liver Cell Atlas: https://singlecell.broadinstitute.org). **(B)** Representative immunofluorescence images of IBA1 (red), NEDD4L (green), and DAPI (white) (Scale bar: 20 μm) in the livers of Oil or CCl_4-treated *Nedd4l*$^{f/f}$ and *Nedd4l*$^{\Delta Mye}$ mice. The number of positive cells was quantified. Values represent mean ± SEM. ****p*<0.001.

Supporting Figure S2

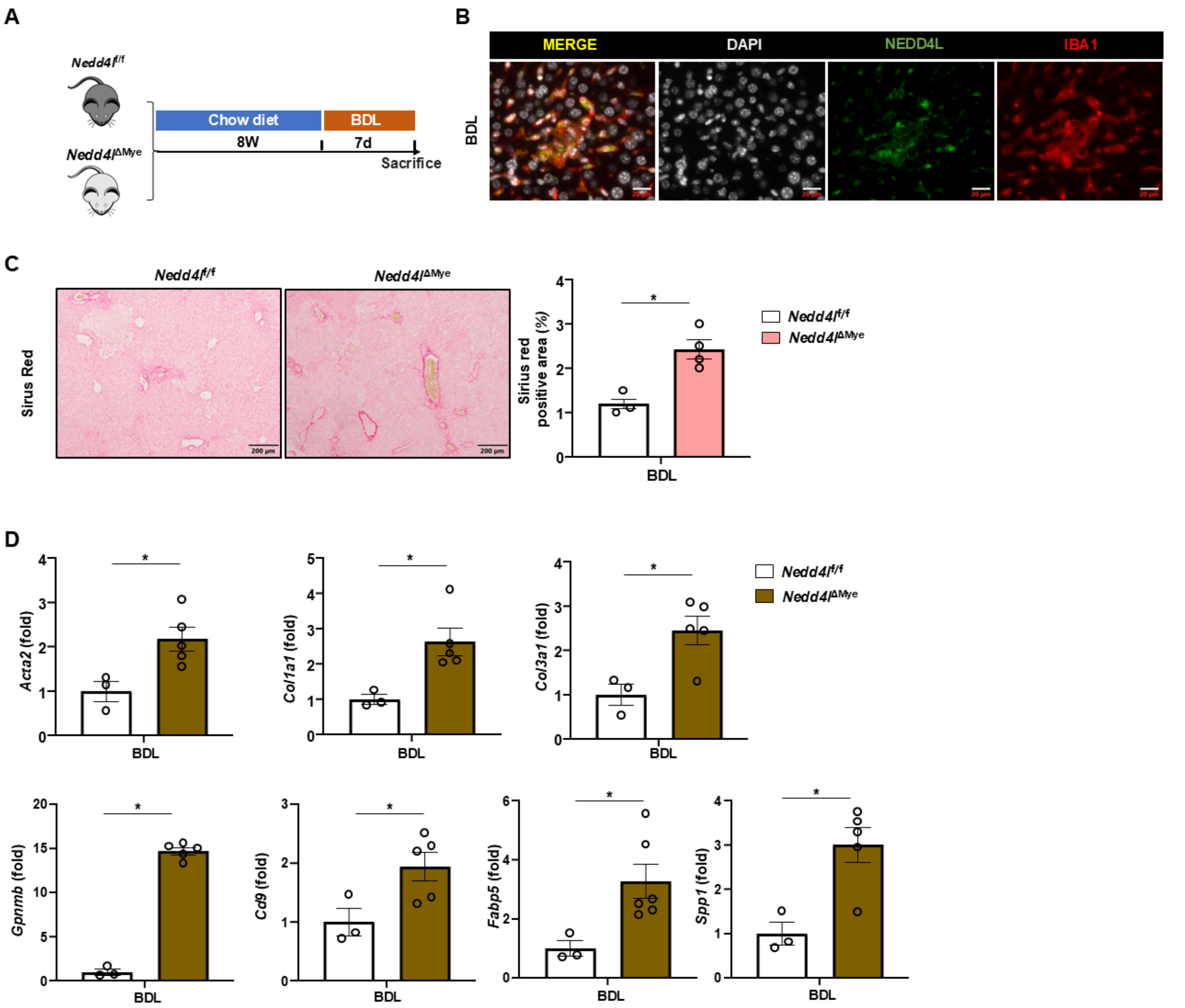

Supporting Figure S2. *Nedd4l* deficiency in macrophages worsens BDL-induced liver fibrosis. **(A)** Eight-week-old male *Nedd4l*$^{\Delta Mye}$ mice and their littermate *Nedd4l*$^{fl/f}$ mice were subjected to bile duct ligation model for 7 days to establish the experimental biliary stasis-related liver fibrosis model. **(B)** Representative immunofluorescence images of IBA1 (red), NEDD4L (green), and DAPI (white) (Scale bar: 20 μm) in the livers of BDL-treated *Nedd4l*$^{f/f}$ mice. **(C)** Representative images of Sirius red staining (Scale bar: 200 μm) from *Nedd4l*$^{f/f}$ and *Nedd4l*$^{\Delta Mye}$ mice are shown. The percentage of positive area was quantified. **(D)** The hepatic expression of *Acta2*, *Col3a1*, *Col1a1*, *Gpnmb, Cd9, Fabp5* and *Spp1* were analyzed by RT-qPCR in liver tissues from *Nedd4l*$^{f/f}$ and *Nedd4l*$^{\Delta Mye}$ mice. Values represent mean ± SEM. *p<0.05.

Supporting Figure S3

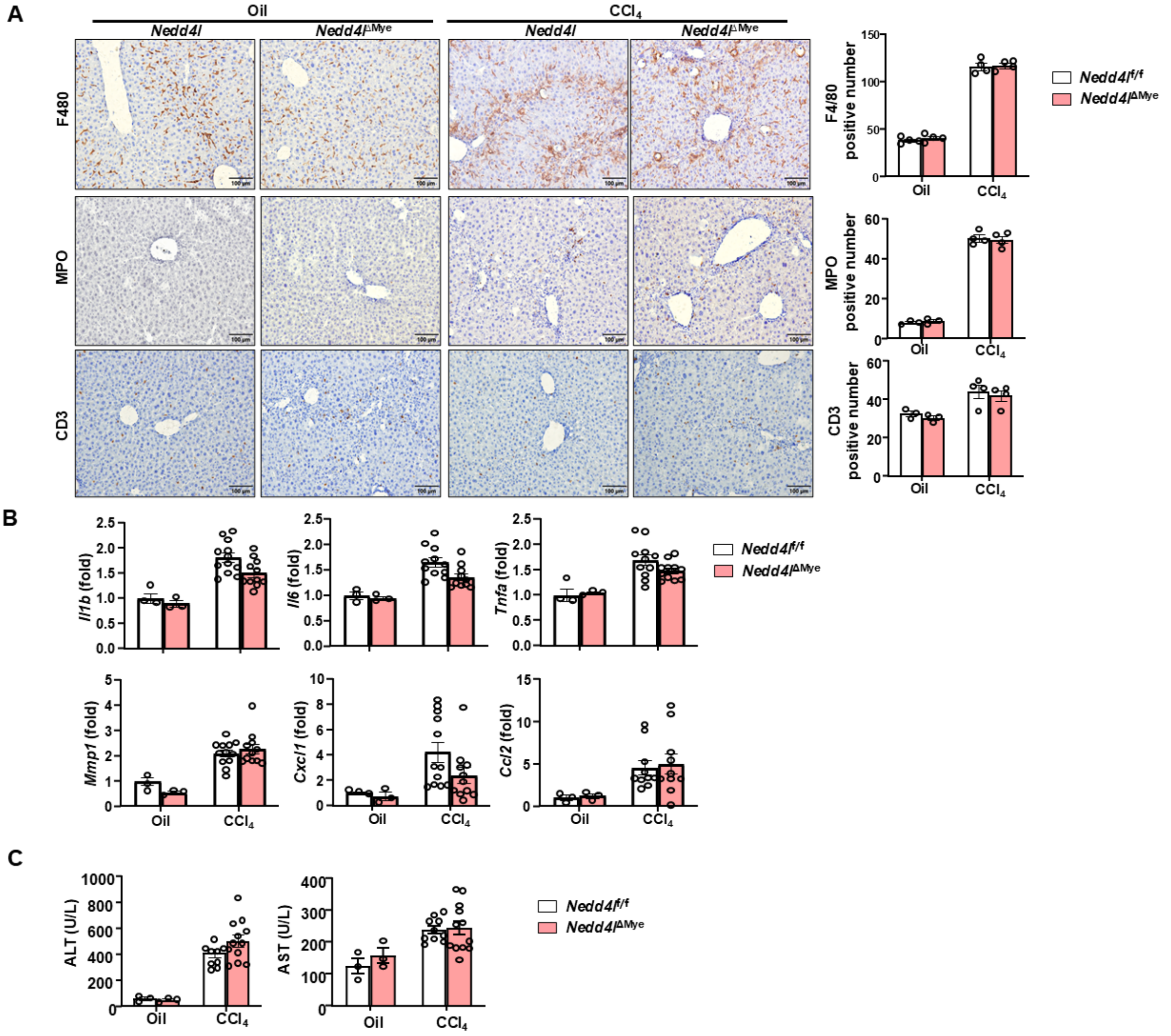

Supporting Figure S3. Myeloid cell-specific knockout of *Nedd4l* does not affect immune cell infiltration and liver injury in CCl_4-induced liver fibrosis. (A) Representative images of F4/80 staining (Scale bar: 200 µm), MPO staining (Scale bar: 200 µm) and CD3 staining (Scale bar: 200 µm) from oil and CCl_4-treated *Nedd4l*$^{f/f}$ and *Nedd4l*$^{\Delta Mye}$ mice are shown. The number of positive cells was quantified. **(B)** The hepatic expression of inflammatory genes was analyzed by RT-qPCR from *Nedd4l*$^{f/f}$ and *Nedd4l*$^{\Delta Mye}$ mice. **(C)** Serum ALT and AST levels were measured. Values represent mean ± SEM.

Supporting Figure S4

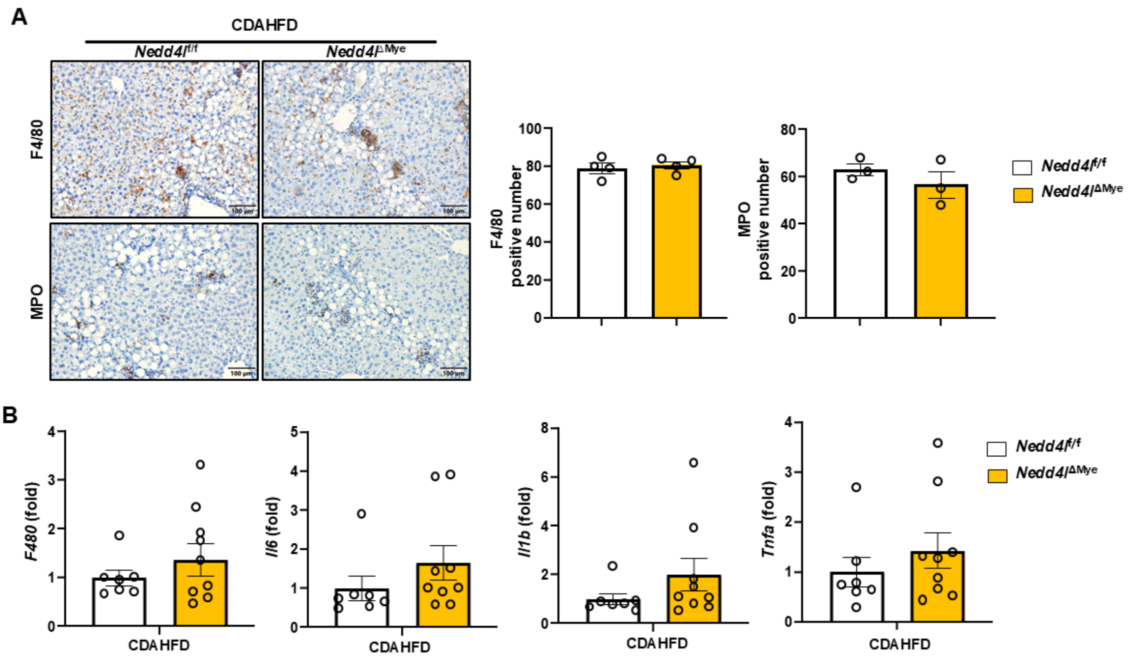

Supporting Figure S4. Myeloid cell-specific knockout of *Nedd4l* does not affect immune cell infiltration in CDAHFD-induced liver fibrosis. (A) Representative images of F4/80 staining (Scale bar: 200 μm), MPO staining (Scale bar: 200 μm) from CDAHFD-fed *Nedd4l*$^{f/f}$ and *Nedd4l*$^{\Delta Mye}$ mice are shown. The number of positive cells was quantified. **(B)**The hepatic expression of inflammatory genes was analyzed by RT-qPCR from *Nedd4l*$^{f/f}$ and *Nedd4l*$^{\Delta Mye}$ mice. Values represent mean ± SEM.

Supporting Figure S5

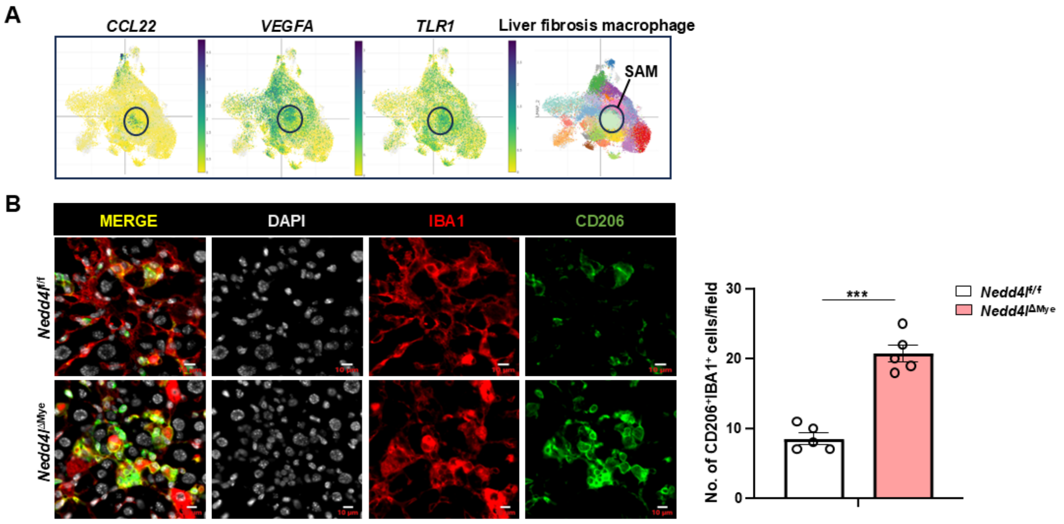

Supporting Figure S5. *Nedd4l* deficiency in macrophage augments profibrotic signaling. (A) Analysis of macrophage *CCL22, VEGFA, TLR1* expression in patients with liver fibrosis by analyzing single-cell sequencing database (Liver Cell Atlas: https://singlecell.broadinstitute.org). **(B)** Representative immunofluorescence images of IBA1 (red), CD206 (green), DAPI (white) (Scale bar: 10 μm) from CCl_4-treated *Nedd4l*$^{f/f}$ and *Nedd4l*$^{\Delta Mye}$ mice are shown. The number of positive cells was quantified. Values represent mean ± SEM. ***p<0.001.

Supporting Figure S6

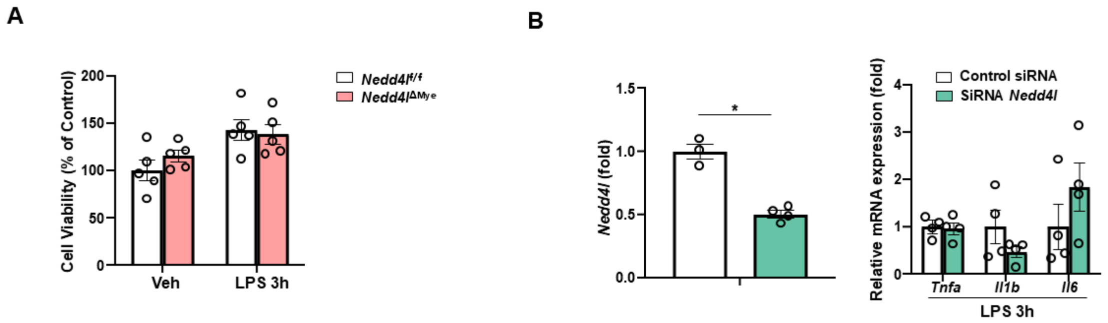

Supporting Figure S6. *Nedd4l* deficiency in macrophages does not affect inflammatory response and cell viability. (A) Bone marrow-derived macrophages (BMDMs) were isolated from *Nedd4l*$^{f/f}$ and *Nedd4l*$^{\Delta Mye}$ mice and stimulated with H_2O (vehicle control) or LPS (100 ng/ml) for 3 hours. Cell viability was assessed using the CCK-8 assay. (B) RAW 264.7 cells were transfected with control siRNA (siCtrl) or Nedd4l-specific siRNA (siNedd4l), and then stimulated with LPS (10 ng/ml) for 3 hours. The knockout efficiency and pro-inflammatory cytokines (*Tnfa*, *Il1b*, and *Il-6*) was analysis by qRT-PCR. Values represent mean ± SEM. *p<0.05.

Supporting Figure S7

A

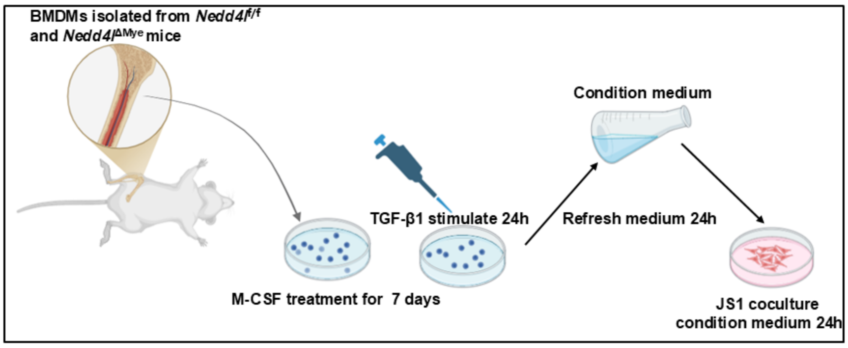

B

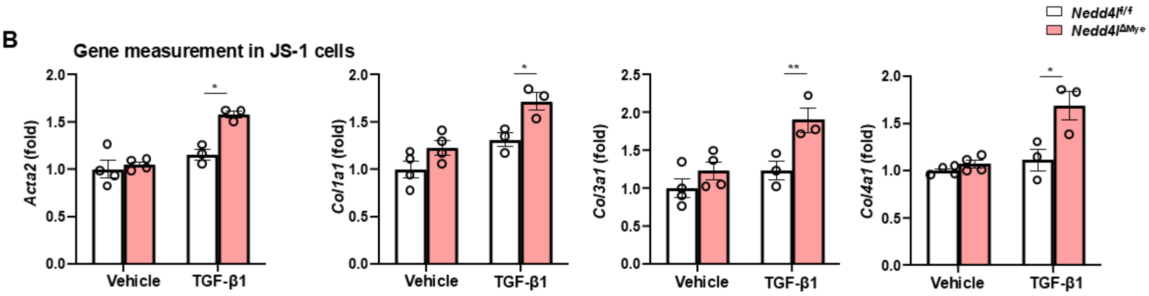

Supporting Figure S7. *Nedd4l* deficiency in macrophages amplifies TGF-β1-driven pro-fibrotic polarization, enhancing the activation of co-cultured JS-1 cells. (A) Schematic diagram of experimental design. BMDMs isolated from *Nedd4l*$^{f/f}$ and *Nedd4l*$^{\Delta Mye}$ mice were stimulated with M-CSF (20 ng/ml) for 7 days, and then stimulated with 10 ng/ml TGF-β1 for 24 hours, followed by removal of the supernatant and further culture in fresh medium for an additional 24 hours. Conditional BMDM medium were co-cultured with JS-1 cells. **(B)** The expression of fibrogenic genes was analyzed by RT-qPCR in JS-1 cells treated with conditional medium from *Nedd4l*$^{f/f}$ and *Nedd4l*$^{\Delta Mye}$ macrophages. Values represent mean ± SEM. *p<0.05, **p<0.01.

Supporting Table S1. The list general characteristics of human samples.

Patient #	Gender	Age	Primary Diagnosis	ALT (IU/L)	AST (IU/L)
1	F	54	steatosis	38	25
2	M	52	steatosis	14	14
3	M	56	MASLD	26	18
4	M	30	MASLD	45	18
5	M	39	MASH, liver fibrosis	92	45
6	M	44	MASH, liver fibrosis	66	36

MASLD: Metabolic dysfunction-associated steatotic liver disease
MASH: Metabolic dysfunction-associated steatohepatitis

Supporting Table S2: The primer sequences for RT-qPCR.

Genes (mouse)	Forward primer (5'-3')	Reverse primer (5'-3')
18s	AACTTTCGATGGTAGTCGCCGT	TCCTTGGATGTGGTAGCCGTTT
Acta2	TCCTGACGCTGAAGTATCCGATA	GGTGCCAGATCTTTTCCATGTC
Col1a1	TAGGCCATTGTGTATGCAGC	ACATGTTCAGCTTTGTGGACC
Col1a2	GGTGAGCCTGGTCAAACGG	ACTGTGTCCTTTCACGCCTTT
Col3a1	TAGGACTGACCAAGGTGGCT	GGAACCTGGTTTCTTCTCACC
Col4a1	CACATTTTCCACAGCCAGAG	GTCTGGCTTCTGCTGCTCTT
Fn1	TTCAAGTGTGATCCCCATGAAG	CAGGTCTACGGCAGTTGTCA
Vimentin	TCCACACGCACCTACAGTCT	CCGAGGACCGGGTCACATA
Tgfb1	CAACCCAGGTCCTTCCTAAA	GGAGAGCCCTGGATACCAAC
Trem2	CTGGAACCGTCACCATCACTC	CGAAACTCGATGACTCCTCGG
Cd9	TGGGATTGTTCTTCGGGTTCC	TCCTTGTGGGTATAGCCCCAG
Spp1	AGCAAGAAACTCTTCCAAGCAA	GTGAGATTCGTCAGATTCATCCG
Gpnmb	AGAAATGGAGCTTTGTCTACGTC	CTTCGAGATGGGAATGTATGCC
Fabp5	AAAGAGCTAGGAGTAGGACTGG	TGTTGCCATCACACGTAATGA
Cd63	GAAGCAGGCCATTACCCATGA	TGACTTCACCTGGTCTCTAAACA
Chil3(Ym1/2)	CAGGTCTGGCAATTCTTCTGAA	GTCTTGCTCATGTGTGTAAGTGA
Ccl22	CTCTGCCATCACGTTTAGTGAA	GTCTTGCTCATGTGTGTAAGTGA
Tlr8	GCCAAACAACAGCACCCAAAT	AGGCAACCCAGCAGGTATAGT
Vegfa	GCACATAGAGAGAATGAGCTTCC	CTCCGCTCTGAACAAGGCT
Arg1	CTCCAAGCCAAAGTCCTTAGAG	AGGAGCTGTCATTAGGGACATC
Nedd4l	GTCCGGCTGTTCCGTACTC	AGGCCATAGTAGGGGTTAAACAT
Timp1	GCAACTCGGACCTGGTCATAA	CGGCCCGTGATGAGAAACT
F480	GGAAAGCACCATGTTAGCTGC	CCTCTGGCTGCCAAGTTAATG
Ly6g	TGCGTTGCTCTGGAGATAGA	CAGAGTAGTGGGGCAGATGG
Il1b	GCAACTCGGACCTGGTCATAA	CGGCCCGTGATGAGAAACT
Il6	TAGTCCTTCCTACCCCAATTTCC	TTGGTCCTTAGCCACTCCTTC
Tnfa	AGGCTGCCCCGACTACGT	GACTTTCTCCTGGTATGAGATAGCAAA
Ccl2	CCAGCCTACTCATTGGGAT	GGGCCTGCTGTTCACAGTT
Cxcl1	ACTGCACCCAAACCGAAGTC	TGGGGACACCTTTTAGCATCTT
Mmp1	CTTCTTCTTGTTGAGCTGGACTC	CTGTGGAGGTCACTGTAGACT

Genes (human)	Forward primer (5’-3’)	Reverse primer (5’-3’)
18S	GGCCCTGTAATTGGAATGAGTC	CCAAGATCCAACTACGAGCTT
ACTA2	GTGACGAAGCACAGAGCAAA	CTTTTCCATGTCGTCCCAGT
COL1A1	CAGATCACGTCATCGCACAA	TGTGAGGCCACGCATGAG
COL3A1	AGGACTGACCAAGATGGGAA	AGGGGAGCTGGCTACTTCTC
COL4A1	CCTTTTGTCCCTTCACTCCA	CTCCACGAGGAGCACAGC
FN1	CGGTGGCTGTCAGTCAAAG	AAACCTCGGCTTCCTCCATAA
TGFB1	CAATTCCTGGCGATACCTCAG	GCACAACTCCGGTGACATCAA
